# CryoEM structure of *Saccharomyces cerevisiae* U1 snRNP offers insight into alternative splicing

**DOI:** 10.1038/s41467-017-01241-9

**Published:** 2017-10-19

**Authors:** Xueni Li, Shiheng Liu, Jiansen Jiang, Lingdi Zhang, Sara Espinosa, Ryan C. Hill, Kirk C. Hansen, Z. Hong Zhou, Rui Zhao

**Affiliations:** 1 Department of Biochemistry and Molecular Genetics, University of Colorado Denver Anschutz Medical Campus, Aurora, CO 80045 USA; 2 Electron Imaging Center for Nanomachines University of California, Los Angeles (UCLA), Los Angeles, CA 90095 USA; 3 Department of Microbiology, Immunology, and Molecular Genetics, UCLA, Los Angeles, CA 90095 USA

## Abstract

U1 snRNP plays a critical role in 5ʹ-splice site recognition and is a frequent target of alternative splicing factors. These factors transiently associate with human U1 snRNP and are not amenable for structural studies, while their *Saccharomyces cerevisiae* (yeast) homologs are stable components of U1 snRNP. Here, we report the cryoEM structure of yeast U1 snRNP at 3.6 Å resolution with atomic models for ten core proteins, nearly all essential domains of its RNA, and five stably associated auxiliary proteins. The foot-shaped yeast U1 snRNP contains a core in the “ball-and-toes” region architecturally similar to the human U1 snRNP. All auxiliary proteins are in the “arch-and-heel” region and connected to the core through the Prp42/Prp39 paralogs. Our demonstration that homodimeric human PrpF39 directly interacts with U1C-CTD, mirroring yeast Prp42/Prp39, supports yeast U1 snRNP as a model for understanding how transiently associated auxiliary proteins recruit human U1 snRNP in alternative splicing.

## Abstract (web summary)

U1 snRNP is critical for 5′ splicing site recognition in pre-mRNA splicing. Here the authors describe the cryo-EM structure of the yeast U1 snRNP and suggest that PrpF39 is an alternative splicing factor essential for the successful recruitment of U1 snRNP by other alternative splicing factors.

## Introduction

Pre-mRNA splicing is catalyzed by the spliceosome, a huge protein–RNA complex composed of the U1, U2, U4, U5, U6 small nuclear ribonucleoprotein complexes (snRNPs) and many non-snRNP related proteins^1^. U1 snRNP, the most abundant snRNP in most eukaryotes^2^, is critical for the initial recognition of 5ʹ-splice site (ss) through the base pairing between a single-stranded region at the 5ʹ-end of U1 snRNA and the 5ʹ-ss^3–6^. Due to its important role in 5ʹ-ss recognition, human U1 snRNP is a frequent target for alternative splicing factors that either facilitate or prevent U1 snRNP from binding to 5ʹ-ss^7^. Several partial structures of human U1 snRNP either reconstituted from individual components or obtained from limited proteolysis of U1 snRNP purified from HeLa cells have been determined with X-ray crystallography^8–10^. The combination of these structures reveals the protein–protein and protein–RNA interaction networks in human U1 snRNP, as well as the structural basis of 5ʹ-ss recognition. Notwithstanding the tremendous value of these structures, many alternative splicing factors loosely associated with human U1 snRNP are not captured by these structures, limiting our understanding of the molecular mechanism of alternative splicing.

On the other hand, the much larger *S. cerevisiae* (we will use “yeast” to specifically represent *S. cerevisiae* throughout) U1 snRNP contains homologs of a number of human alternative splicing factors as stable components (Supplementary Table 1 and Supplementary Fig. 1). Specifically, while purified human U1 snRNP (~250 kD) contains only ten proteins (seven Sm proteins, U1-70K, U1A, U1C), purified yeast U1 snRNP (~800 kD) contains seven additional stably associated proteins (Luc7, Nam8, Prp39, Prp40, Prp42, Snu56, and Snu71)^11, 12^. Of these additional proteins, Nam8, Prp40, Luc7, and Snu71 have human homologs (TIA1, PRPF40, Luc7L, RBM25) that are weakly associated with human U1 snRNP and are implicated in alternative splicing^13, 14^. We will use U1 core to refer to the ten yeast proteins common between yeast and human, and auxiliary proteins to refer to the seven additional yeast proteins. Furthermore, the yeast U1 snRNA (568 nucleotides (nt)) is 3.5 times the size of its human counterpart (164 nt)^15, 16^. Taken together, the more complex yeast U1 snRNP may provide a valuable model for understanding the structural basis of U1-mediated alternative splicing in higher eukaryotes.

Here, we report the structure of yeast U1 snRNP at 3.6 Å resolution determined by cryo electron microscopy (cryoEM). This structure provides a framework to integrate a wealth of existing genetic and biochemical data on yeast U1 snRNP as well as to understand the structure and function of human auxiliary proteins, offering new insight into the molecular mechanism of alternative splicing.

## Results

### Overall structure of yeast U1 snRNP

**Fig. 1:**
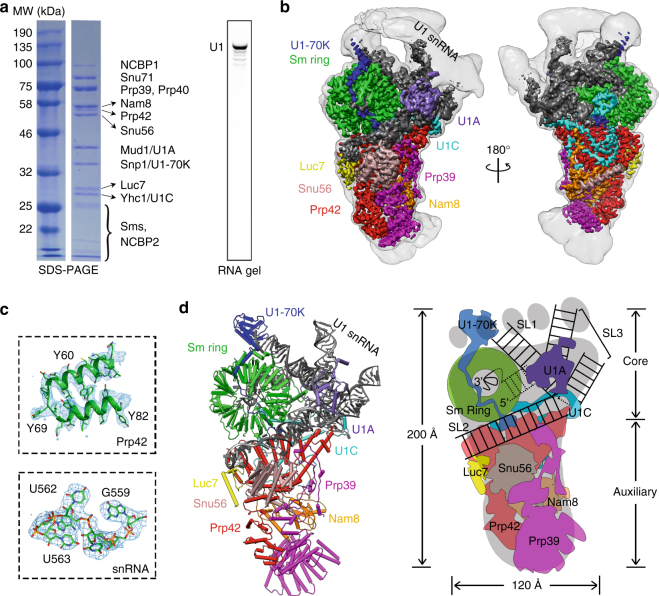
Overall cryoEM structure of the yeast U1 snRNP. **a** Purified yeast U1 snRNP sample used for cryoEM structure determination was analyzed on SDS-PAGE and its protein components identified using mass spectrometry analyses. The sample was also analyzed using solution hybridization^48^ to demonstrate the presence of U1 snRNA in the sample. **b** Surface representation of the cryoEM map of yeast U1 snRNP. The density map (3.6 Å resolution) for each component is shown in different colors. The EM map is low-pass filtered to 8 Å resolution to show more flexible regions of the U1snRNP (transparent gray). **c** Examples of the 3.6 Å cryoEM density with atomic models built in. **d** Model of the yeast U1 snRNP with each protein in a different color. A schematic representation of the structure is also shown using the analogy of a foot. All figures in the paper are prepared using Chimera^32^

We purified U1 snRNP from yeast through a TAP-tag^17^ on U1A. Purified yeast U1 snRNP contains all known protein components and the U1 snRNA^11, 12^ (Fig. 1a, Supplementary Table 1). It also contains NCBP1 and NCBP2, subunits of the nuclear cap-binding protein complex that has been shown to interact with U1 snRNP^18, 19^. We determined the cryoEM structure of yeast U1 snRNP to an overall resolution of 3.6 Å with local resolutions in the central regions reaching 3.0–3.5 Å (Fig. 1b, Supplementary Figs. 2–5, Supplementary Movie 1). Most secondary structural elements are well defined and a large percentage of amino-acid side chains and RNA bases are identifiable (Fig. 1c, Supplementary Figs. 6–8). We built atomic models for almost all essential domains of U1 snRNA (excluding the 5ʹ-end 10 nt of 5ʹ-ss recognition sequence and ~230 nt non-essential region) and partial or complete models for 15 of the 17 proteins (Fig. 1d, Supplementary Table 1, Supplementary Fig. 9). The modeled yeast U1 snRNP has a dimension of ~200 × 120 × 80 Å with the overall shape resembling a foot in three dimensions (Fig. 1d). The U1 core (Sm ring, U1-70K, U1C, U1A, and U1 snRNA) is located in the “ball-and-toes” region while all modeled auxiliary U1 snRNP proteins are located in the “arch-and-heel” region. Of these auxiliary proteins, the Prp42/Prp39 paralogs^20^ form a central scaffold connecting auxiliary U1 snRNP proteins to the core.

### U1 snRNA forms a platform for 5′ ss recognition

**Fig. 2:**
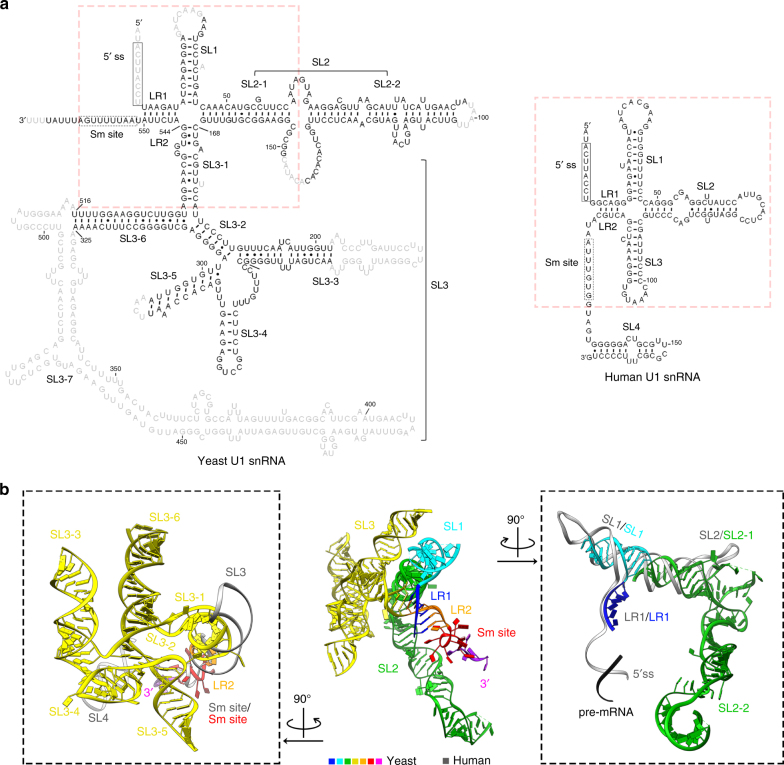
The similarities and differences between human and yeast U1 snRNA. **a** Secondary structures of human and yeast U1 snRNA based on the three dimensional structures. Light gray nucleotides are not built in the structure. Red dashed box represents the core components common between the two RNAs. **b** Structure of the entire yeast U1 snRNA (LR1, SL1, SL2, SL3, LR2, Sm site, and 3ʹ-tail in blue, cyan, green, yellow, orange, red, and purple, respectively) in an orientation similar to the right panel of Fig. 1b. Also shown are structural comparisons of isolated areas of human (grey, PDB ID 3PGW) and yeast U1 snRNA including the SL3 to the 3ʹ-tail (left) and 5ʹ-ss-binding region to SL2 (right)

U1 snRNA is located in the “ball-and-toes” region of U1 snRNP (Fig. 1d). From the 5ʹ to 3ʹ-end, it can be divided into the 5ʹ-ss recognition sequence, long-range interaction 1 (LR1), Stem-Loop (SL) 1, SL2, SL3 (further divided into SL3-1 to SL3-7), LR2, the Sm-binding site, and the 3ʹ-tail, consistent with previous models derived from secondary structure prediction and phylogenic comparison^16, 21, 22^ (Fig. 2a). The yeast U1 snRNA structure overlaps with its human counterpart in the LR1, LR2, SL1, and Sm-binding site regions, while the SL2, SL3, and 3ʹ-tail regions are drastically different between the two RNAs. With the exception of the Sm-binding site that is buried within the channel formed by the seven Sm proteins, the U1 snRNA is mostly surface exposed, typically with one side of the RNA interacting with proteins and the other side facing the solvent. We will discuss below the common features between yeast and human U1 snRNA, followed by discussions of SL2 and SL3.

U1 snRNA has identical sequences in its first 10 nt between human and yeast (Fig. 2a). In the human U1 snRNP structure, nucleotides 3–10 of U1 snRNA basepair with the AG/GUAAGU sequence of the pre-mRNA (/designates the 5ʹ-ss)^9, 10^. In yeast U1 snRNA, the first ten nucleotides are not modeled but there is a stretch of weak density near nucleotide 11 that likely corresponds to the 5ʹ-end of U1 snRNA (Supplementary Fig. 10a). The region (nt 11–16) immediately downstream of the 5ʹ-ss recognition site basepairs with nucleotides (nt 545–550) immediately upstream of the Sm-binding site, forming LR1 and LR2, respectively (Fig. 2b, middle). A similar pair of long-range interactions exist in the human U1 snRNA^9^. Following LR1, U1 snRNA in yeast forms SL1 located in a similar position as the human SL1 (Fig. 2b, right), which is the U1-70K-binding site in both species. The similar positions of LR1, LR2, and SL1 in both species suggest that that yeast U1 snRNA likely basepairs with pre-mRNA in a comparable manner to the human U1 snRNA for 5ʹ-ss recognition.

Yeast SL2 is roughly three times the size of human SL2, and can be divided into two sub-domains. The first sub-domain (SL2-1) consists mainly of a stem formed between nt 46–60 and 154–167 that superimposes well with SL2 in human U1 snRNA (Fig. 2b, right). The second sub-domain (SL2-2) consists mostly of nt 69–95 basepaired with 103–132, which is oriented nearly perpendicular to the first sub-domain and does not have a counterpart in human U1 snRNA (Fig. 2b, right). SL2-2 interacts with Prp42 and Snu56, possibly strengthening the connection of auxiliary U1 snRNP proteins with the core. The lack of SL2-2 in human U1 snRNA may be partially responsible for the weak association between human U1 snRNP and auxiliary proteins.

Yeast SL3 is about 15 times the size of human SL3, forming multiple stem loops (SL3-1 to SL3-7) (Fig. 2b, left). Other than half of SL3-3, the tip of SL3-5, and SL3-7 which cannot be modeled due to weak density, the secondary structure of yeast U1 snRNA is in agreement with previous predictions^16, 21, 22^. SL3 largely resides on the periphery (“toes”) of yeast U1 snRNP with some interactions with SmB and U1C, possibly explaining why it can be deleted with limited effect on growth^21^.

### Comparison of the yeast and human U1 snRNP core

**Fig. 3:**
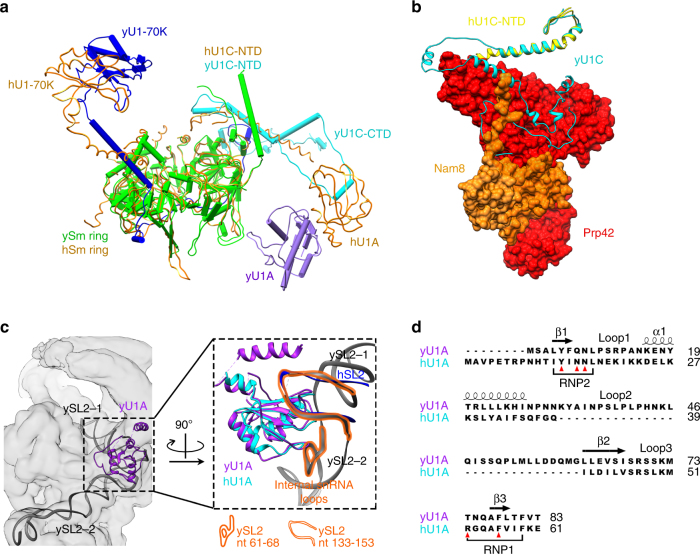
The similarities and differences between yeast and human U1 snRNP core. **a**. The core protein components between human and yeast U1 snRNP are organized similarly. All human U1 components are colored in orange (PDB ID 3PGW and 3CW1), while yeast Sm ring, U1-70K, U1C, and U1A are colored in cyan, blue, green, and purple, respectively. The two U1 snRNPs are superimposed through their Sm rings. U1A and U1-70K positions are slightly shifted. U1C-NTDs overlap, while the U1C-CTD is not visible in the human U1 snRNP structure. **b** Yeast U1C-NTD (green) superimposes well with that of human U1C (yellow, PDB ID 3CW1). The C-terminal region of yeast U1C interacts extensively with Prp42 (red) and to a lesser extent with Nam8 (orange). **c** Yeast U1A (purple) binds to the internal loop region (nt 61–68 and nt 133–153) between SL2-1 and SL2-2 (black). In the inset panel, the human U1A RRM1 (cyan, PDB ID 1OIA) structure is superimposed on yeast U1A RRM1 (purple). The β-sheet face of yeast U1A RRM1 binds to the internal loop (nt 133–153, highlighted in orange) located in a similar position as the RNA (dark blue) that human U1A binds. The yeast U1A also binds to the second internal loop (nt 61–68, highlighted in orange) through multiple loops in its RRM1 domain. **d** Sequence alignment between human and yeast U1A RRM1 demonstrates differences between residues in RNP1 and RNP2 that are known to bind RNA in human U1A (red triangles) and the long loop 2 in yeast U1A

The architecture of the yeast U1 snRNP core and the entire human U1 snRNP share much similarity. When the yeast and human Sm rings are superimposed, the N-terminal domains (NTD) of U1C proteins are located at the same positions, and yeast U1-70K (Supplementary Fig. 10b) and U1A are in the general vicinity of their human counterparts with an 18 and 26 Å positional shift in their centers of mass, respectively (Fig. 3a). The NTD of yeast U1C (residues 1–48) has a conformation similar to its human counterpart (Fig. 3a, b). Yeast U1C likely binds the U1 snRNA and pre-mRNA duplex in a manner comparable to human U1C, considering the similar conformation and spatial position between yeast and human U1C NTD and the U1 5ʹ-ss-binding region. Like the human U1-70K, yeast U1-70K wraps around the Sm ring with a long N-terminal arm that contacts and stabilizes U1C, followed by a RRM domain near SL1 (Fig. 3a). The similar spatial organization of the U1 core in both species highlights a conserved mechanism for 5ʹ-ss recognition.

Despite the conserved spatial organization between the yeast and human U1 cores, there are several unique features in yeast U1C and U1A that have functional implications. Yeast U1C (231 residues) is largely composed of loops with one dominant helix in its NTD and another one in the C-terminal domain (CTD) (Fig. 3b). While the N-terminus of U1C is well conserved between human and yeast, their C-termini are not^23^. Yeast U1C-CTD (not built in the human structure) makes contacts with SmD3, Nam8, and interacts extensively with Prp42 (Fig. 3b). The interaction between U1C-CTD and Prp42 will be described in detail in the Prp42/Prp39 section below, and this interaction is likely a major factor for retaining Prp42 in the yeast U1 snRNP.

Yeast U1A is positioned near the 90-degree bend between the two SL2 sub-domains (Fig. 3c). Similar to human U1A, the yeast U1A protein contains two RRM domains and RRM1 can be modeled into the cryoEM density. The yeast U1A sequence diverges from its human counterpart (most notably a 30-residue insertion between α1 and β2 in yeast U1A^24, 25^) and was shown to bind a different consensus sequence (CACAUAC) in an internal loop of SL2^25, 26^. In our structure, the β-sheet face of yeast U1A RRM1 binds to the internal loop encompassing nt 133–153 (containing the CACAUAC sequence) located at the junction of SL2-1 and SL2-2. Other than contributions from residues in the RNP1 an RNP2 motifs, which demonstrate some differences between yeast and human (Fig. 3d), the long insertion between α1 and β2 in yeast U1A (Fig. 3d) contributes significantly to the interaction with this loop. These two factors potentially explain why the consensus U1A-binding sequence in yeast is different from human. In addition, the β1/α1, β2/β3, and α2/β4 loops are all involved in extensive interaction with the other internal loop (nt 61–68) at the junction of SL2-1 and SL2-2. It has been postulated that the non-essential yeast U1A functions to stabilize the active U1 snRNA conformation^24^. The extensive interaction between the yeast U1A RRM1 and two internal loops located at the junction of SL2-1 and SL2-2 places U1A at a perfect position to stabilize the bent conformation of SL2 of U1 snRNA.

### Prp42/Prp39 connect auxiliary proteins to the core

Prp42 (544 residues) is a central component of yeast U1 snRNP that interacts with all visible auxiliary proteins, and connects them to the U1 core in the “ball-and-toes” region. The high quality of the Prp42 density allowed us to model essentially the entire Prp42 (residues 2–540). Prp42 contains multiple tetratricopeptide repeats (TPR), a 34-residue motif that form a pair of antiparallel α helices A and B^27^. Tandem TPRs form a right-handed helical solenoid-like structure, which is often involved in protein–protein interactions^27^. Prp42 contains a roughly perpendicular N-terminal domain with four TPRs and a C-terminal domain with seven TPRs that are connected by a ~100-residue linker composed of four helices.

**Fig. 4:**
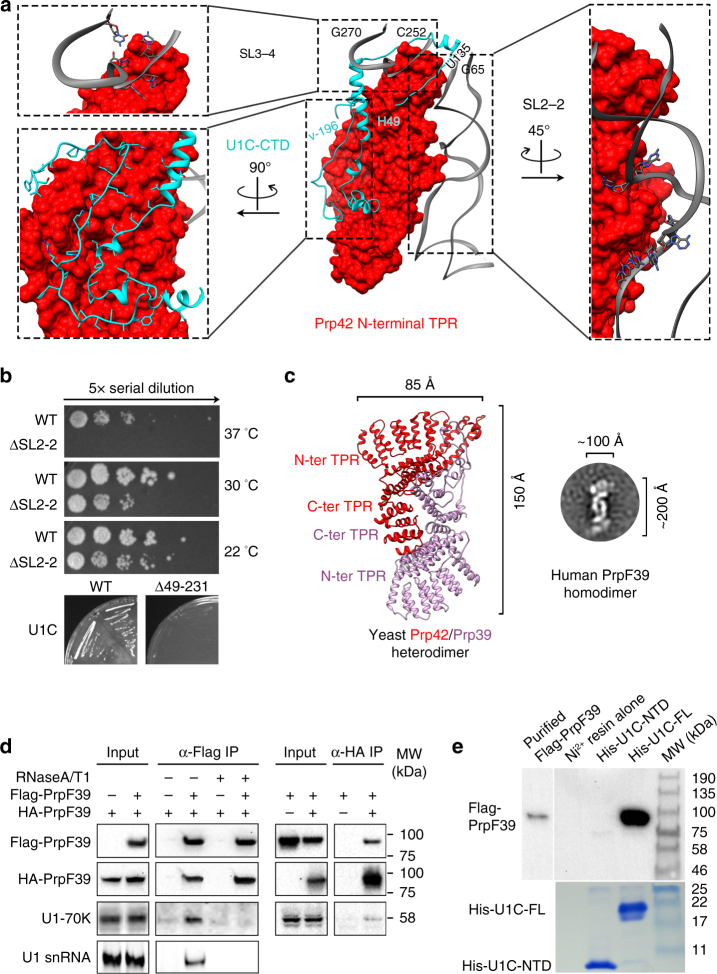
Structures and interactions of yeast auxiliary proteins Prp42, Prp39, and human PrpF39. **a** The N-terminal TPR domain of Prp42 interacts extensively with the C-terminal domain of U1C, and to a lesser extent, with SL3-4 and SL2-2 of U1 snRNA. Details of the specific residues involved in the Prp42-U1C interaction are presented in Supplementary Table 2. **b** Yeast strain carrying ΔSL2-2 shows slightly less robust growth than WT at 30 and 22 °C and is inviable at 37 °C. Yeast strain carrying U1C-CTD deletion is inviable when WT U1C is shuffled out on a FOA plate. **c** Prp39 and Prp42 have a similar overall structure and interact with each other through their C-terminal TPR domains (left). A representative 2D class average of negative-stained images of purified human PrpF39 has dimensions and shape highly similar to the Prp42/Prp39 heterodimer (right). **d** Western blot and solution hybridization analyses demonstrate that IP using anti-HA antibody pulls down HA-PrpF39 and Flag-PrpF39 (with or without RNase A/T1 treatment), as well as human U1-70K and U1 snRNA (only in the absence of RNase A/T1 treatment) from HEK293 cells co-transfected with HA-PrpF39 and Flag-PrpF39 (left). Pull-down with anti-Flag antibody shows the same result (right, not treated with RNase or probed for U1 snRNA). **e** Pull-down using purified human U1C FL or NTD (residues 1–61) demonstrates that U1C FL but not the NTD binds to purified Flag-PrpF39. The top portion is a western blot using an anti-Flag antibody and the bottom is a SDS-PAGE gel stained with Coomassie blue to show U1C protein levels

In addition to interacting with all visible auxiliary proteins (Prp39, Nam8, Snu56, Luc7), Prp42 interacts extensively with the U1C-CTD (Fig. 4a). The N-terminal TPR domain of Prp42 forms a shallow binding groove that accommodates U1C, similar to how other TPR-containing proteins interact with their protein partners^27^. There are 64 pairs of residues interacting with each other (Supplementary Table 2) encompassing 5322 Å^2^ of buried surfaces between Prp42 and U1C (calculated using Cocomaps^28^).

Prp42 also interacts with SL3-4 (798 Å^2^ buried surface area) and SL2-2 (1450 Å^2^ buried surface area) (Fig. 4a), although not as extensively as with U1C. The interaction between Prp42 and SL3-4 is mostly mediated between five nucleotides in the bulge at the base of SL3-4 and six residues on the loops of the helices connecting the N- and C-terminal TPR domains (Supplementary Table 3). Most of the Prp42/SL2-2 interaction is formed between residues in the N-terminal TPR domain and the backbone of SL2-2 (Supplementary Table 3). It is worth noting that protein HCF107 contains 11 HAT (Half-A-TPR, a variant of TPR) repeats that interact with a single-stranded 11 nt RNA in a sequence specific manner, although the nature of residues on HCF107 that contribute to this binding is unclear^29^. The interaction between the N-terminal TPR domain of Prp42 and the backbone of the double-stranded region of SL2-2 reveals a new mode of how TPR-containing proteins can bind RNA, expanding the role of TPR domains beyond the typical protein–protein interaction mediators. In addition to TPR proteins, many other helical repeat proteins have been shown to bind either double-stranded DNA (dsDNA) or single-stranded RNA (ssRNA)^30, 31^, although binding to dsRNA has not yet been observed, making the interaction between Prp42 and SL2 unique among helical proteins that recognize nucleic acids (Supplementary Fig. 11).

We next used truncation analyses to evaluate the functional significance of Prp42 interaction with U1 snRNA and U1C-CTD. Deletion of large regions of SL3 containing SL3-4 (e.g., nt 192–507) has no apparent growth phenotype at 30 °C^21^. We then deleted SL2-2 from U1 snRNA (U1-ΔSL2-2) and residues 49–231 from U1C (U1C-ΔC). A yeast strain carrying U1C-ΔC as the only U1C is inviable at 30 °C (Fig. 4b). A yeast strain carrying U1-ΔSL2-2 as the only U1 snRNA shows slightly less robust growth at 30 and 22 °C than WT, and does not grow at 37 °C (Fig. 4b), possibly because the higher temperature significantly destabilizes the association of auxiliary proteins with the U1 core without SL2-2. These growth phenotypes are consistent with the notion that the interaction between Prp42 and U1C (and to a lesser extent between Prp42 and SL3-4/SL2-2) likely play an important role in connecting the auxiliary proteins to the U1 core.

Prp39 (629 residues) has a strong sequence homology with Prp42 (22% sequence identity and 50% similarities) and the two are considered paralogs arisen from the gene duplication event in yeast^20^. Not surprisingly, Prp39 has a very similar overall structure to Prp42 with an additional ~70-residue C-terminal extension (Fig. 4c, Supplementary Figs. 10b and 12a). The C-terminal TPR domains of Prp42 and Prp39 interact extensively, both using the face made of helix A, to form a heterodimer-like structure (Fig. 4c, left). Although we have not proven that Prp39 and Prp42 form a standalone heterodimer in cells, we use the term “heterodimer” to reflect the extensive interaction between Prp39 and Prp42 observed in the context of yeast U1 snRNP structure.

### Homodimeric human PrpF39 directly interacts with U1C-CTD

Although only Prp39 has a reported human homolog PrpF39, we note that both Prp42 and Prp39 share sequence similarities to human PrpF39 (17% identity and 31% similarity with Prp42; 18% identity and 33% similarity with Prp39), which seems to be the only human protein that has significant sequence similarities to Prp39 and Prp42. An intriguing possibility derived from this observation is that there are two copies of PrpF39 forming a homodimer in human U1 snRNP. To test this hypothesis, we co-transfected a Flag-tagged PrpF39 and a HA-tagged PrpF39 into HEK293 cells. We demonstrate that immunoprecipitation (IP) of Flag-PrpF39 brings down HA-PrpF39 in western blot and vice versa with or without RNase treatment (Fig. 4d), supporting our hypothesis. PrpF39 also pulls down U1-70K and U1 snRNA only in the absence of RNase treatment (Fig. 4d), indicating that PrpF39 associates with U1 snRNP but not directly through U1-70K. Furthermore, the representative two-dimensional (2D) class average of negative-stained images of purified PrpF39 has a dimension and shape that is strikingly similar to the Prp42/Prp39 heterodimer (Fig. 4c, right; Supplementary Fig. 12b). The volume of the preliminary three-dimensional (3D) model of PrpF39 (~220 × 10^3^ Å^3^, calculated using Chimera^32^) is also nearly identical to that of the Prp42/Prp39 heterodimer (~210 × 10^3^ Å^3^). Consistent with a PrpF39 homodimer formed through its CTD, purified PrpF39 treated with BS3 and mass spectrometry analyses revealed crosslinked peptides exclusively within PrpF39-CTD, including identical peptides crosslinked to itself through the same amino acid (Supplementary Table 4). In addition, we illustrate that human U1C full-length (FL) protein expressed and purified from *E. coli* directly interacts with purified Flag-PrpF39 in a pull-down assay, while U1C-NTD (residue 1–61) does not (Fig. 4e). These results demonstrate that human PrpF39 forms a homodimer that directly interacts with the U1C-CTD, mirroring the interaction of Prp42/Prp39 heterodimer with U1C-CTD in yeast U1 snRNP.

### Meiotic regulators Nam8 and Snu56 interact with Prp42/Prp39

**Fig. 5:**
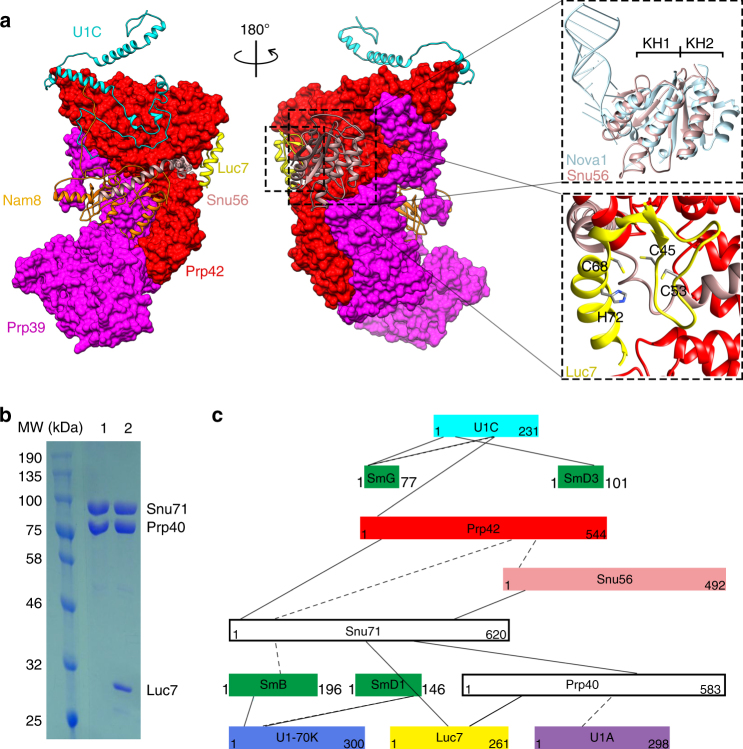
Structures and interactions of yeast auxiliary proteins Nam8, Snu56, and Luc7. **a** The relative positions of Nam8, Snu56, and Luc7 with respect to Prp42, Prp39, and U1C. Snu56 has two KH-like domains that can be superimposed very well with the two KH domains in Nova1 (PDB ID 2 ANR). Luc7 contains a pseudo zinc-finger and it interacts with Prp42 and Snu56. **b** Snu71 + Prp40 (lane 1) or Snu71 + Prp40 + Luc7 (lane 2) can be co-expressed in yeast and purified as a complex using the protein A-tag on Prp40. **c** Mass spectrometry analyses of purified U1 snRNP crosslinked with BS3 reveal crosslinked peptides between proteins. Solid lines represent confidently identified crosslinks with a false discovery rate of less than 5%. Dashed lines represent crosslinks identified with a false discovery rate of 5–20% and are considered probable. Only inter-molecular crosslinks are shown for clarity

Nam8 (523 residues) is dispensable for mitotic growth but required for the splicing of several meiotic genes by recruiting U1 snRNP to weak splicing sites, providing an example of splicing regulation in yeast^33–35^. Nam8 contains three RRM domains, flanked by an N-terminal leader peptide and a C-terminal tail^35^. The C-terminal half of Nam8 (the linker between RRM2 and RRM3, RRM3, and the C-terminal tail) can be modeled into the cryoEM density and is nestled between the Prp42/Prp39 paralogs (Fig. 5a). RRM3 of Nam8 has the typical RRM-fold with its helical face binding to Prp42/Prp39, an interaction mode frequently used by RRM domains to bind their protein partners^36^. The β-sheet face is occluded by the C-terminal tail of Nam8 and a C-terminal helix of Prp39, making RRM3 inaccessible to pre-mRNA. This observation, in combination with the fact that RRM1 is dispensable for Nam8 function^35^, makes RRM2 the most likely candidate to bind meiotic-specific pre-mRNAs and help recruit U1 snRNP. We use the term “recruit” in the broad sense to also include scenarios where alternative splicing factors help strengthen or stabilize interactions between U1 snRNP and weak 5ʹ-ss. The Ser/Asn/Gln-rich Nam8 linker between RRM2 and RRM3 interacts with U1C through a number of Asn and Gln residues, although this interaction (2356 Å^2^ buried surface) is less extensive than that between Nam8 and Prp42/Prp39 (7120 Å^2^ buried surface).

TIA1, the human homolog of Nam8 and an alternative splicing factor, also has three RRM domains^14, 37^ and binds to the U-rich region downstream of a weak 5ʹ-ss^14, 37^. RRM3 domain of TIA1 is thought to be necessary and sufficient for interactions with in vitro translated human U1C from rabbit reticulocyte extract^14^. The possibility remains that the association of TIA1 RRM3 with human U1C is not direct but mediated through other factors in the extract (such as the Prp39 homolog), similar to RRM3 of yeast Nam8. Other biochemical and structural studies suggest that RRM3 is important for RNA binding^37, 38^. However, these studies use isolated RRM3 domain or RRM2-3 domains without either the C-terminal region of TIA1 or other proteins (such as Prp39), which could occlude the RNA-binding surface of RRM3 as we observed in yeast U1 snRNP and change the RNA-binding properties of TIA1 in the cell. These studies also demonstrate that RRM1 does not contribute to RNA binding and RRM2 has the highest RNA-binding affinities among the RRM domains, in line with the RNA-binding mode suggested by our yeast U1 snRNP structure.

Snu56 (492 residues) is dispensable for the splicing of several mitotic genes investigated but is required for meiotic splicing^39^. The N-terminal half of Snu56 can be modeled in our structure and it wraps around the junction between the N-terminal and C-terminal TPR domains of Prp42 (Fig. 5a). Snu56 contains two previously unrecognized consecutive KH-like domains, a widespread RNA-binding module, flanked by an N-terminal helix and two C-terminal helices. The two KH-like domains are packed almost identically to the KH1/HK2 domains of Nova-1 (Fig. 5a), forming a pseudodimer of KH domains through inter-domain interactions^40^. KH2 of Snu56 interacts with Prp42, similar to KH2 of Nova-1 that interacts with other proteins. Although KH1 of Snu56 does not contain the canonical RNA-binding motif of KH domains, we cannot rule out that KH1 of Snu56 binds RNA using different residues, analogous to the KH1 domain of Nova-1 that binds RNA. The two C-terminal helices of modeled Snu56 wrap around Prp42 and the last helix interacts with RRM3 of Nam8, which is located on the opposite face of Prp42 from the KH domains of Snu5. Snu56, therefore, acts like a “clamp” which uses its C-terminal helix and KH domains to clamp the RRM3 domain of Nam8 onto Prp42. Snu56 could potentially affect the splicing of meiotic genes through its own RNA-binding activity, or by contributing to the association of Nam8 to U1 snRNP through its clamp-like structure.

### Auxiliary proteins Luc7, Prp40, and Snu71 form a trimer

Luc7 (261 residues) contains two putative Zn-finger domains^12^, and the first one can be modeled into the density that contacts both Prp42 and Snu56 (Fig. 5a). Residue C45 is proposed to be part of the Zn-finger but is too far from the other two Cys (C53 and C68) and one His (H72) to coordinate Zn^2+^ (Fig. 5a), making this region a pseudo Zn-finger. Prp40 was shown to interact with both Luc7 and Snu71 through its FF domain^41^. Indeed, our co-expression experiments demonstrate that both Prp40 + Snu71 + Luc7 and Prp40 + Snu71 form a stable complex that can be purified using the protein A-tag on Prp40 (Fig. 5b). There is a large but not well-defined density surrounding Luc7, which could accommodate the remaining Luc7, Snu71, and Prp40 proteins (Supplementary Fig. 10c). Consistent with the above observation, purified U1 snRNP treated with BS3 and subsequent mass spectrometry analyses identified crosslinked peptides within Luc7-Snu71, Luc7-Prp40, Prp40-Snu71, Snu71-Prp42, and Snu71-Snu56 (Fig. 5c, Supplementary Table 5). Human homologs of Luc7 (Luc7L), Snu71 (RBM25), and Prp40 (PrpF40) have been implicated in alternative splicing regulation^13, 42, 43^. These proteins may also interact with each other to form a trimer and associate with human U1 snRNP through Luc7L and PrpF39.

## Discussion

Our yeast U1 snRNP structure demonstrates that the core components common between yeast and human are organized almost identically, in spite of the dramatically larger yeast U1 snRNP. Although the yeast SL2 and SL3 regions are almost three and 15 times the size of their human counterparts, SL2 forms a 90° bend in the middle and SL3 is largely peripheral, enabling the U1 core proteins to bind at similar spatial positions as their human counterparts. This similarity highlights a conserved mechanism of 5ʹ-ss recognition by U1 snRNP from yeast to human.

In contrast to these similarities, yeast U1 snRNP is much more complex than the human U1 snRNP with seven additional stably associated proteins, all of which are essential (Nam8 is dispensable for mitotic growth but required for meiosis). One possibility is that these auxiliary proteins are essential for the stability of the U1 core. However, depletion of Prp42 produced a smaller U1 snRNP that is fully functional when micrococcal nuclease-treated WT extract was added, suggesting that U1 snRNP without Prp42 is stable^20^. Barring undiscovered vital functions not related to splicing, the essential nature of these auxiliary proteins suggests that there likely exist genes in yeast whose 5ʹ-ss recognition and splicing are dependent on these auxiliary proteins in addition to the U1 core. These genes, even if there are only a few, may be essential for the viability of yeast. The splicing of these genes are likely constitutively required instead of being regulated, potentially explaining why yeast U1 snRNP has evolved to stably incorporate these auxiliary proteins instead of having them loosely associated to allow regulation.

Although it has long been postulated that the much larger yeast U1 snRNA provides binding surfaces for many yeast auxiliary U1 snRNP proteins, SL2-2 and SL3-4 seems to be the only snRNA domains that forms substantial interactions with the auxiliary proteins (mostly Prp42). However, because Prp42 interacts extensively with U1C and contacts SmD3 and SmG, the interactions between Prp42 and these snRNA domains are unlikely to be required for connecting the auxiliary proteins to the U1 core, but rather may have been evolved to maximize the stable association of auxiliary proteins. This proposition is consistent with the mild growth phenotype in ΔSL2-2 (Fig. 4b) and ΔSL3-4 strains^21^. Along the same line, any potential Prp42 homolog in human will likely be able to associate with U1 snRNP, but with a weaker affinity due to the lack of SL2-2 and SL3-4 in human. The function of most of the much longer and more complex SL3 is unclear. One possibility is that SL3 may provide binding sites for protein factors that play a role in the splicing of specific pre-mRNAs under special conditions. This would explain why most of SL3 can be deleted with limited effect on growth in standard lab conditions^21^.

**Fig. 6:**
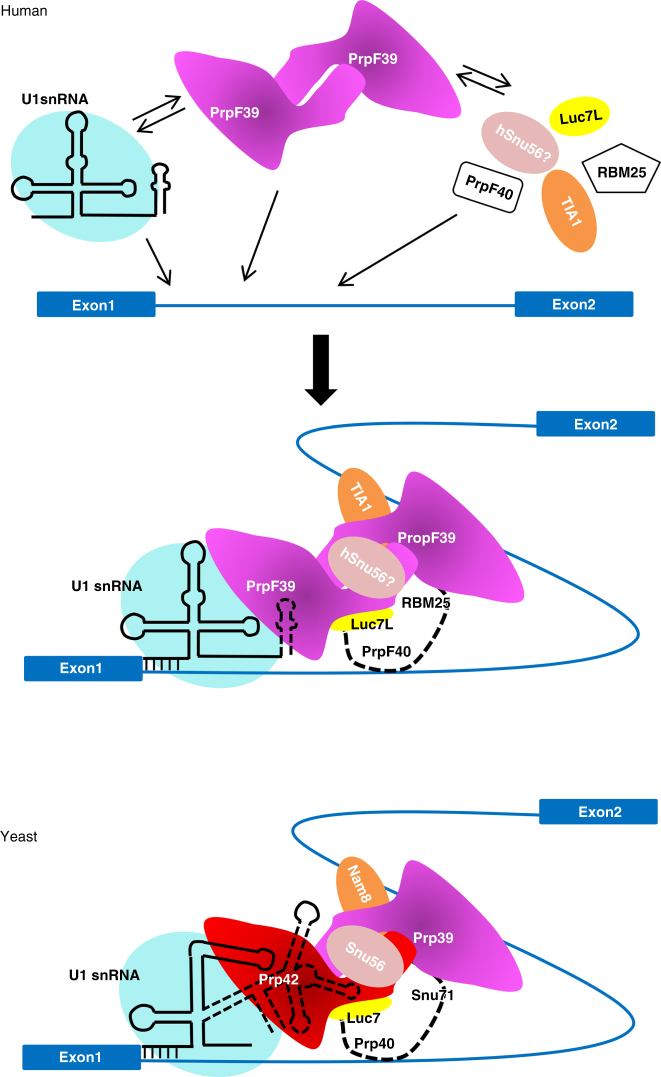
Yeast U1 snRNP serves as a model for the association of alternative splicing factors with human U1 snRNP. This model is supported by our observation that human PrpF39 forms a homodimer that directly interacts with U1C-CTD, mirroring the interaction between the Prp42/Prp39 paralogs and U1C-CTD in yeast U1. In human cells, an alternative splicing factor such as TIA1, Luc7L, PrpF40, and RBM25 (and possibly a functional homolog of Snu56) binds pre-mRNA through their RNA-binding domains and likely interact with the U1 snRNP in the same fashion as in the yeast U1 snRNP to facilitate the binding between U1 snRNA and 5ʹ-ss. Although some of these alternative splicing factors can directly interact with core components of U1 snRNP, PrpF39 likely plays a major role in facilitating the association between these factors and the core U1 snRNP, possibly through its extensive interactions with these factors, analogous to those observed in the yeast U1 snRNP structure. PrpF39 may also serve as an alternative splicing factor on its own through possible interactions between its N-terminal TPR domain and pre-mRNA

The more complex yeast U1 snRNP serves as a valuable model for understanding the molecular details of how the human or other mammalian U1 auxiliary proteins bind and recruit U1 snRNP in alternative splicing (Fig. 6), which has been difficult to capture due to the weak nature of these interactions. The power of yeast U1 snRNP as a model is demonstrated by its prediction of the structure and function of PrpF39, a scarcely studied protein, that is confirmed by our biochemical analyses. In yeast U1 snRNP, the Prp42/Prp39 paralogs form a central scaffold connecting auxiliary proteins to the core. Both proteins have similar levels of sequence homology to a single human protein PrpF39, leading us to hypothesize that PrpF39 exists as a dimer in human cells. Our pull-down and EM analyses confirmed that human PrpF39 exists as a homodimer that directly interacts with U1C through its CTD, mirroring the interaction between the Prp42/Prp39 heterodimer with U1C-CTD in yeast. The PrpF39 homodimer also draws an interesting analogy to two other proteins involved in mRNA processing (SART3 which is the human functional homolog of yeast Prp24 and mouse CstF77 required for 3ʹ-end processing) that contain the HAT repeat. Both proteins contain a N-terminal and a C-terminal HAT domain that are nearly perpendicular to each other^44, 45^. Each protein forms a homodimer through interactions between its C-terminal HAT domains^44, 45^. The human PrpF39 homodimer may adopt asymmetry as observed in other homodimers^46, 47^, allowing the asymmetric binding of the U1 core, auxiliary proteins, and pre-mRNA to the PrpF39 dimer. Alternatively, even if PrpF39 exists as a symmetric homodimer, a population of the dimer could have the U1 core bound on one monomer and pre-mRNA (or an auxiliary factor) on the other.

The structure of yeast U1 snRNP leads us to further hypothesize that PrpF39 functions as an alternative splicing factor. We have demonstrated that the N-terminal TPR domain of Prp42 can bind RNA (SL3-4 and SL2-2), and the HAT domain has been shown to bind ssRNA in a sequence specific manner^29^. The N-terminal TPR domain of PrpF39, if arranged in the same fashion as Prp39 in yeast U1 snRNP, would be completely solvent-exposed and in a perfect position to bind sequence-specific RNA to recruit U1 snRNP to 5ʹ-ss. Furthermore, all modeled yeast auxiliary proteins interact extensively with Prp42/Prp39, and Prp42 interacts with U1C-CTD to connect the yeast auxiliary components to the U1 core. This central scaffolding role of Prp42/Prp39 suggests a key function of PrpF39 in U1-mediated alternative splicing events in human. Although some alternative splicing factors have been observed to directly interact with core components of U1 snRNP, this interaction may not be sufficient. We suggest that the successful recruitment of U1 snRNP to 5ʹ-ss by many alternative splicing factors would depend on human PrpF39, suggesting that modulation of PrpF39 levels could have profound effects on alternative splicing events mediated through U1 snRNP.

The yeast U1 snRNP structure also provides specific and testable predictions on how known alternative splicing factors (such as TIA1 and Luc7L) interact and recruit the human U1 snRNP. An intriguing observation from the yeast U1 snRNP structure is that the binding of auxiliary proteins to Prp42/Prp39 (and consequently to the U1 core) is not mutually exclusive. Although human alternative splicing factors have mostly been studied in isolation, it is foreseeable that two or more alternative splicing factors may bind the human U1 snRNP simultaneously and influence alternative splicing choices of pre-mRNAs with binding sites for multiple factors. The effect of expressing two or more alternative splicing factors on the splicing profile in cells may be different from expressing only one, adding an additional layer of fine tuning in the regulation of alternative splicing.

## Methods

### U1 snRNP purification

Twelve liters of U1A/Mud1-TAP tagged yeast cells were cultured in a modified YEPD medium (2xYEP + 6% d-Glucose) at 30 °C to an OD_600_ of 10. The cell pellets (~150 g) were re-suspended in 40 ml of lysis buffer (50 mM Tris-HCl, pH 8.0, 150 mM NaCl, 0.05% NP-40, 0.2 mM EDTA). The cell suspension was snap-frozen into liquid nitrogen to form yeast “popcorn” and cryogenically ground using a SPEX 6870 Freezer/Mill. The frozen cell powder was thawed at room temperature and re-suspended in an additional 240 ml of lysis buffer with protease inhibitor cocktails (Roche) and 1 mM Benzamidine. The cell lysate was first centrifuged at 27,845×*g* for 1 h in a GSA rotor (Sorvall) and the supernatant was further centrifuged at 167,424×*g* rpm in a 45Ti rotor (Beckman) for 1.5 h at 4 °C. The supernatant was incubated with 3 ml of IgG Sepharose-6 Fast Flow resin (GE Healthcare) overnight at 4 °C. The resin was washed with IgG washing buffer (10 mM Tris-HCl, pH 8.0, 150 mM NaCl, 0.05% NP40, 0.5 mM dithiothreitol (DTT), 1 mM benzamidine and protease inhibitor cocktails), and incubated with TEV protease in 1.5 ml TEV150 buffer (10 mM Tris-HCl, pH 8.0, 150 mM NaCl, 0.02% NP40, 0.5 mM DTT, and 0.2 mM EDTA). About 2 ml of eluate was subsequently applied to 10–30% v/v glycerol gradient centrifuged at 159,642×*g* at 4 °C in a SW40 rotor for 21 h. The fractions from the gradient were analyzed by both sodium dodecyl sulfate polyacrylamide gel electrophoresis (SDS-PAGE) and solution hybridization^48^ with an IRDye-700 labeled U1-specific primer. The peak fractions were combined and supplemented with 2 mM CaCl_2_ and incubated with 300 μl calmodulin affinity resin (Stratagene) overnight at 4 °C. The resin was washed with washing buffer (20 mM Hepes7.9, 150 mM NaCl, 1 mM MgCl_2_, 2 mM CaCl_2_, 1 mM imidazole), and eluted six times with 300 μL eluting buffer (20 mM Hepes pH 7.9, 150 mM NaCl, 1 mM MgCl_2_, 2 mM EGTA) each time in a column by gravity flow. After confirming that the eluate contains all known U1 snRNP proteins and U1 snRNA using SDS-PAGE and mass spectrometry (Supplementary Table 1), the eluate with the optimal concentration was used for sample preparation for cryoEM imaging.

### Negative-stain electron microscopy (EM)

An initial 3D model was generated using negative-stain EM and random conical tilt (RCT) reconstruction^49^. Briefly, 2 μl of sample was loaded onto a glow-discharged carbon film grid which was then stained with 0.8% (w/v) uranyl formate. Negative-stain EM micrographs were collected at a nominal magnification of 70,000× on an FEI Tecnai T20 using SerialEM^50^. For each target of interest on the grid, two micrographs were recorded with the grid tilted at 65° and 0°, respectively. A total number of 727 pairs of micrographs were acquired. CTFFIND4^51^ was used to determine the defocus values for un-tilted micrographs, while CTFTILT^52^ was used for tilted micrographs. Corresponding particles from tilt pairs were picked using a modified version of TiltPicker^53^ and extracted into 100 × 100 pixel images (pixel size of 4.29 Å). Each particle image was corrected for contrast transfer function (CTF) by phase-flipping with the corresponding defocus and astigmatism values using Bsoft^54^. The particles from un-tilted micrographs were subjected to reference-free 2D classification using EMAN^55^. Particles in good classes with well-defined and interpretable features were selected for further processing using SPIDER^56^. We then generated 3D reconstructions from the tilted particle images using the RCT algorithm in SPIDER. Lastly, the good un-tilted particle images selected from the previously mentioned 2D classification were used to refine the 3D reconstruction using the projection-matching algorithm in EMAN^55^ (Supplementary Fig. 2).

For negative-stain image analyses of PrpF39, a total of 506 micrographs were collected at a nominal magnification of 70,000× on an FEI Tecnai T20. The defocus value of each micrograph was determined by CTFFIND4. A total of 185,966 particles were automatically picked without reference using Gautomatch (developed by Kai Zhang, MRC Laboratory of Molecular Biology, Cambridge, UK). The particles were boxed out in dimensions of 84 × 84 square pixels (pixel size of 4.29 Å), and then subjected to reference-free 2D classification using GPU accelerated RELION2-beta^57^.

### CryoEM sample preparation and data collection

For cryoEM, 2.5 μl of sample was applied onto a glow discharged Quantifoil (R1.2/1.3, 400 mesh) holey grid. The grid was blotted and flash-frozen in liquid ethane with an FEI Vitrobot Mark IV whose sample chamber was at room temperature and 100% humidity. CryoEM grids were loaded into an FEI Titan Krios electron microscope operated at 300 kV for automated imaging using Leginon^58^. A total number of 4934 dose-fractionated micrographs were acquired at a nominal magnification of 105,000× using a Gatan K2 Summit camera operating in counting and super-resolution modes attached to a Gatan GIF Quantum LS Imaging Filter (using a slit width of 20 eV) (Supplementary Fig. 3). The dose rate was set to eight counts per physical pixel per second on the camera. A total number of 48 frames were acquired for each micrograph with a 0.25 s exposure time for each frame. Dose-fractionated frames were aligned for drift correction using the GPU-accelerated program MotionCor2^59^. The first frame was skipped during drift correction. Two averages, one with dose weighting and the other without dose weighting, were generated for each micrograph after drift correction. The averaged micrographs were 2× binned to yield a calibrated pixel size of 1.36 Å for the following image processing. The averages without dose weighting were used only for defocus determination and those with dose weighting were used for all other steps of data processing.

### CryoEM image processing

The defocus value of each cryoEM micrograph was determined by CTFFIND4, generating values ranging from −1.6 to −4.0 μm. Initially, a total of 971,568 particles were automatically picked from 4184 micrographs without reference using DoGpicker^53^. The particles were boxed out in dimensions of 320 × 320 square pixels and binned to 160 × 160 square pixels (pixel size of 2.72 Å) before further processing by the GPU accelerated RELION2-beta. Several iterations of reference-free 2D classification were subsequently performed to remove ice, contaminants, and bad particles (i.e., classes with fuzzy or un-interpretable features), yielding 382,062 good particles. The previously refined RCT model was low-pass filtered to 60 Å to serve as an initial model for 3D classification. The 3D classification generated three classes, and only one class showed features of intact U1 snRNP structure. Particles from this class were subjected to 3D auto-refinement, resulting in a 6.3 Å map.

The 6.3 Å map was low-pass filtered to 20 Å to generate a set of projections, which served as the reference for automatic particle picking using Gautomatch. A total of 1,759,402 particles were picked from all data sets comprising 4934 micrographs. The particles were boxed out and binned to 160 × 160 square pixels (pixel size of 2.72 Å) before further processing. After one round of 3D classification with five classes, only one class containing 47.2% of all particles showed features corresponding to intact U1 snRNP. We re-centered these particles and removed duplications based on the unique index of each particle given by RELION. In all, 534,641 particles (30.4% of all particles) were generated and subjected to reference-free 2D classification, resulting in 435,376 good particles (24.7% of all particles). These particle images were then un-binned to 320 × 320 square pixels (pixel size of 1.36 Å) and used for 3D auto-refinement with RELION2-beta. A soft mask was applied during the auto-refinement to exclude the highly flexible parts of the particles. The resulting reconstruction has an overall resolution of 3.6 Å, which shows clear secondary structure elements for both proteins and RNA. Finally, the 435,376 good particles were subjected to another round of 3D classification with the same mask. This yielded 352,900 particles (20.0% of all particles) in three good classes. Auto-refinement of particles combined from these three classes yields a map with an average resolution of 3.6 Å. This map shows better secondary structure elements and amino-acid side chains than the previous 3.6 Å map in most regions and was selected as the final map for subsequent model building.

All resolutions reported above are based on gold-standard FSC 0.143 criterion^60^. FSC curves were calculated using soft spherical masks and high-resolution noise substitution was used to correct for convolution effects of the masks on the FSC curves^61^. Prior to visualization, all maps were corrected for the modulation transfer function of the detector, and then sharpened by applying a negative B-factor, which was estimated using automated procedures^62^. Local resolution was estimated using ResMap^63^. A flow chart depicting the above image processing process and overall quality of the map are presented in Supplementary Figs. 4 and 5. Data collection and reconstruction statistics are presented in Supplementary Table 6.

### Model building

The protein and RNA components of the final model are listed in Supplementary Table 1. Initially, the Sm ring with its characteristic shape was identified and was docked into the map. The location of the Sm ring in combination with the known architecture of human U1 snRNP helped us identify the location of U1-70K, U1C zinc finger, U1A, and the four-way junction of U1 snRNA. The well-defined density in this region allowed us to build atomic models for seven Sm proteins, N-terminal U1-70K (residues 3–91), and U1C (residues 3–47). Two extend stretches of density with characteristics of tandem helix pairs were then assigned to Prp42 and Prp39, which are both TPR (tetratricopeptide repeat)-containing proteins. Much of the central region of their density map is resolved at resolutions better than 3.6 Å (ranging from 3.2–3.6 Å, Supplementary Fig. 5c). The well-defined density of this area allowed us to build atomic models for the full length of Prp42 and C-terminal TPR domain of Prp39 (residues 326–552). Although Prp42 and Prp39 share 50% sequence similarities, 50% of the sequences is completely different between the two proteins, including many residues in the TPR motifs of Prp42 that are different from their counterparts in Prp39. For example, residues 60 to 69 are YSSMLNEFPY in Prp42 (Supplementary Fig. 6a) and their corresponding residues are WQ-ILRKYPL in Prp39. Some other prominent differences include: Prp39 has a long C-terminal tail (residues 568–620, Supplementary Fig. 6b) that does not exist in Prp42 and Prp42 has an extra loop region (residues 194–230) absent in Prp39. These differences and the high-quality density map in the Prp42/Prp39 region enabled us to unambiguously differentiate these two proteins. A RRM-fold-like density was identified as the third RRM domain of Nam8 (residues 303–383). A remaining density region close to RNA was identified as the N-terminal domain of Snu56 (residues 47–254). After the model building process described above, there still existed some well-defined density on the surface of the map, which was clearly not the RNA component. These regions were first traced with polyalanine. Subsequent sequence analysis of their surrounding proteins and unidentified components enabled us to unambiguously identify the C-terminal region of U1C (residues 48–195), the inter-RRM linker of Nam8 (residues 289–302), the C-terminal region of Prp39 (residues 561–627), and a long helix following the N-terminal domain of Snu56 (residues 255–303). The models were verified by side chain densities and secondary structure prediction results.

The above model building process in U1 snRNP was facilitated by published structures and mass spectroscopy analysis of the crosslinked U1 snRNP sample. The atomic models of U1-70K and U1C were produced by CHAINSAW^64^ using structures of their human homologs. The homology models for Prp42 and Prp39 were generated by I-TASSER server^65^, which were fitted into the density by CHIMERA^32^, and manually rebuilt using COOT^66^. Secondary structure predictions also greatly helped the model building process. For Nam8 and Snu56, polyalanine α-helices and β-sheets were first traced into the density based on their secondary structure predictions by PSIPRED server^67^. Unassigned loop regions and side chains were rebuilt using COOT. Sequence assignment was mainly guided by bulky residues, such as Trp, Tyr, Phe, and Arg. Other residues including Gly and Pro also helped the assignment process. Unique patterns of sequences were utilized for validation of residue assignment.

The double-helix structures of RNA could be easily seen from the EM density map. As indicated above, the four-way junction of U1 snRNA could be unambiguously identified. To avoid the mis-assignment of the RNA component, we first focused on building the atomic model of the proteins. After that, we were able to assign the density for Stem Loop 1 and Stem Loop 2 based on the positions of U1-70K and U1A. The position of Stem Loop 3 was subsequently identified from the EM map. The RNA building process was facilitated by the predicted secondary structure of yeast U1 snRNA^16, 68^, different sizes of purine and pyrimidine bases, and reported binding site preferences of RNA-binding protein. The RNA sequences were manually built into the density using COOT, and further adjusted using RCrane^69^ and ERRASER^70^.

The model building process described above was mainly used for central regions of U1 snRNP with resolution between 3.2–5.0 Å (Supplementary Fig. 5c). Resolution for the periphery of the complex was more varied, ranging from 6 Å to 12 Å. The EM density of these regions is insufficient to build the atomic model (Supplementary Fig. 5c). Focused classification of the U1-70K RRM domain and Prp39 N-terminal domain improved the density enough to enable docking of a homology model of these proteins (U1-70K: 94–188; Prp39: 43–285). The homology models were obtained using I-TASSER server and fitted into the density using CHIMERA^32^. A summary of the final models built for each U1 snRNP component is listed in Supplementary Table 1.

The model was refined using PHENIX in real space^71^ with secondary structure and geometry restraints. The final models were refined using REFMAC in reciprocal space with secondary structure restraints generated in PROSMART and RNA restraints (basepairing and stacking) generated in LIBG^72^. Over-fitting of the overall model was monitored by refining the model in one of the two independent maps from the gold-standard refinement (Supplementary Fig. 5d). Refinement statistics of U1 snRNP were summarized in Supplementary Table 6. Protein structures built de novo were individually validated by Morprobity scores^57^, Ramachandran plots and EMRinger scores^73^ (Supplementary Table 7). The structure of yeast U1 snRNA was directly validated by the Molprobity server (Supplementary Table 8). Representative densities for regions of yeast U1 auxiliary proteins, U1 snRNP core, and RNA are shown in Supplementary Figs. 6–8, respectively.

### Crosslinking and mass spectrometry

Purified U1 snRNP was crosslinked with 100 μM BS3 for 30 min at room temperature, then the reaction was quenched by adding 50 mM Tris-HCl, pH 8.0. Crosslinked samples were proteolytically digested with trypsin as previously described^68^. Briefly, ~50 μg of crosslinked sample was reduced, alkylated, and digested at 1:50 with sequencing grade trypsin (Promega) by incubating at 37 °C for 18 h. Tryptic digests were then acidified to 0.1% formic acid and resulting peptides were desalted by solid phase extraction over a C18 spin-tip (Pierce). Peptides were concentrated and acetonitrile removed by vacuum centrifugation and then brought up to final volume for analysis by mass spectrometry.

Crosslinked peptides were then analyzed by nano-UHPLC-MS/MS (Easy-nLC1000, QExactive HF, Thermo Fisher Scientific). For sample injection, 16 µl of sample was directly loaded onto an in-house packed 100 µm i.d. × 250 mm fused silica column packed with Synergi Hydro C16 resin (4 µm, 80 Å, Phenomenex). Samples were run at 350 nL/min over a 90 min linear gradient from 5–32% acetonitrile with 0.1% formic acid. The mass spectrometer was operated in positive ion mode with a precursor scan range of 400–2000 m/z, followed by stoichiometric sampling of the top 20 most intense precursor ions for collision induced dissociation at a collision energy of 35 eV. Data acquisition was performed using Xcalibur (version 4.0) software.

Instrument raw files were converted to de-isotoped, centroided peak lists using in-house script (PAVA, UCSF). Generated peak lists were searched against eighteen proteins making up the U1snRNP from *S. cerevisiae* of the Swiss-prot database (update 2017_01_24) using StavroX (v3.6.0.1)^74^. Search parameters included carbamidomethylation-C as a fixed modification, oxidation-M and BS3 as variable modifications, allowing for two missed cleavages. Precursor mass tolerance was set to 3 ppm, with MS/MS mass tolerance set to 10 ppm. Results were manually validated and visualized using both xVis and ProXL^75, 76^.

### U1 snRNA and U1C deletion and growth phenotype analyses

We generated yeast U1 snRNA ΔSL2-2 deletion from pSE538 Snr19^21^ (gift of C. Guthrie) using inverse PCR amplification and the NEBuilder HiFi DNA Assembly kit (NEB). The plasmids were transformed into yeast strain yJU46 [*MATα, his3, trp1, lys2, ade2, snr19::LYS2, (U1 WT, URA3 CEN ARS)*]^21^ (gift of C. Guthrie), and wild-type U1 snRNA was replaced by plasmid shuffling. Fivefold serial dilutions of each strain were spotted on YEPD plates and incubated at indicated conditions for growth phenotype analyses. We used a similar strategy to generate yeast U1C deletion for growth phenotype analyses. The wild-type U1C plasmid p413-YHC1 and *yhc1Δ* [p316-YHC1] yeast strains^77^ are gifts of Dr. Beate Schwer.

### Pull-down assays

For the PrpF39 pull-down experiment, 293FT cells were transfected with pEF-Bos-Flag-PrpF39 and/or pEF-Bos-HA-PrpF39 plasmids using calcium phosphate. Transfected HEK293FT cells were harvested and lysed in lysis buffer containing 50 mM Hepes, pH 7.5, 150 mM NaCl, 1.5 mM MgCl_2_, 5 mM KCl, 0.1% NP40, 1 mM DTT, and protease inhibitor cocktails. Cell lysates were centrifuged at 18,000×*g* for 30 min at 4 °C and supernatants were incubated with anti-Flag M2 agarose beads (Sigma) or HA antibody immobilized on protein G beads (Roche) for 3 h at 4 °C. Beads were washed with cell lysis buffer twice. Beads were incubated with 100 µl lysis buffer containing 0.5 U RNase A and 20 U RNase T1 (Life Technologies, AM2286) or no RNase, at room temperature for 20 min. Beads were then washed with cell lysis buffer another six times. Half of beads were boiled with 2× SDS-sample buffer for SDS-PAGE and western blot (Supplementary Fig. 12c). The other half of beads were digested by proteinase K and detected using IRDye-700 labeled DNA oligo that hybridizes specifically to human U1 snRNA.

We also purified Flag-PrpF39 proteins from the HEK293FT cells transfected with pEF-Bos-Flag-PrpF39 using anti-Flag M2 agarose beads. We eluted the protein from resin using 3× Flag peptide. The proteins were further purified by gel filtration using a Superose 6 Increase 10/300 GL column (GE Healthcare). The purified protein was used for negative-stain EM analysis and U1C pull-down experiment.

For the human U1C pull-down experiment, we expressed His-tagged full-length human U1C and U1C-NTD (residues 1–61) in *E. coli* and purified the proteins on nickel resin (Invitrogen). After washing the resin three times with the lysis buffer (50 mM Tris-HCl pH 7.5, 500 mM NaCl, 5 mM imidazole, 10% glycerol, and 1 mM DTT), we increased imidazole to 20 mM and washed the resin another three times. Nickel resin (5 μl) with or without U1C proteins was incubated with 1.5 μg purified Flag-PrpF39 protein in the binding buffer (50 mM Hepes pH 6.9, 50 mM NaCl, 4 mM Cysteine, 0.2% dimethyl sulfoxide) at 4 °C for 2 h. The resin was then wash three times using 500 μl of washing buffer (20 mM Tris-HCl pH8.0, 50 mM NaCl, 1 mM DTT), transferred to a new tube, and washed another two times. Proteins were eluted off the resin using the 25 μl of elution buffer (20 mM Tris-HCl pH8.0, 50 mM NaCl, 500 mM imidazole, 1 mM DTT) and run on SDS-PAGE for Coomassie stain and western blot.

### Prp40-Snu71-Luc7 and Prp40-Snu71 purification

The coding regions of yeast Prp40, Snu71, and Luc7 were amplified by PCR using genomic *S. cerevisiae* DNA as templates, and ligated into pRS414, pRS416, and pRS317 vectors, respectively, each between a GPD promoter and a CYC 1 terminator. Yeast strain BCY123 was transformed with different combinations of the plasmids and cultured in the appropriate synthetic drop-out media. The protein complexes were purified using the 2× ProtA tag fused to Prp40 by IgG Sepharose-6 Fast Flow resin and released by TEV protease. The proteins were further purified by gel filtration using a Superose 6 Increase 10/300 GL column.

### Data availability

The atomic coordinates have been deposited in the PDB with ID 5UZ5 and the EM density has been deposited to EMD with ID EMD-8622. All data supporting the findings of this study are available within the article and its Supplementary Information files, or from the authors upon request.

## Electronic supplementary material

Supplementary Information

Description of Additional Supplementary Files

Supplementary Movie 1
