# Author Correction: CryoEM structure of *Saccharomyces cerevisiae* U1 snRNP offers insight into alternative splicing

**DOI:** 10.1038/s41467-018-03708-9

**Published:** 2018-04-11

**Authors:** Xueni Li, Shiheng Liu, Jiansen Jiang, Lingdi Zhang, Sara Espinosa, Ryan C. Hill, Kirk C. Hansen, Z. Hong Zhou, Rui Zhao

**Affiliations:** 1 Department of Biochemistry and Molecular Genetics, University of Colorado Denver Anschutz Medical Campus, Aurora, CO 80045 USA; 2 Electron Imaging Center for Nanomachines, University of California, Los Angeles (UCLA), Los Angeles, CA 90095 USA; 3 Department of Microbiology, Immunology and Molecular Genetics, UCLA, Los Angeles, CA 90095 USA

Correction to: *Nature Communications* 10.1038/s41467-017-01241-9, published online 19 October 2017

The originally published version of this Article contained several errors in Figure 2, panel a: the basepair register in SL3-4 of yeast U1 snRNA was depicted incorrectly; the basepair for A287-U295 in yeast U1 snRNA was erroneously present; basepairs for U84-G119, G309-U532, A288-U295 and U289-A294 in yeast U1 snRNA were missing; the bulging nucleotide in SL3 of human U1 snRNA was depicted as G instead of C; and the dashed boxes defining the 5′ ss binding site and Sm site in both human and yeast snRNAs were not drawn accurately. These have now been corrected in both the PDF and HTML versions of the Article.

