# Supplementary material for: CryoEM structure of Saccharomyces cerevisiae U1 snRNP offers insight into alternative splicing
Source: Nat Commun. 2017 Oct 19;8:1035. doi: 10.1038/s41467-017-01241-9 (PMC5648754; doi:10.1038/s41467-017-01241-9)
Supplement: Supplementary file 1 — Supplementary Information [file 41467_2017_1241_MOESM1_ESM.pdf]

Supplementary Fig. 1

```
Prp42             1 --------------------------------------------------      0
                    
PrpF39            1 MQNSHMDEYRNSSNGSTGNSSEVVVEHPTDFSTEIMNVTEMEQSPDDSPN     50

Prp42             1 -------------MDKYTALIHDE-----NFSTLTLNVSRYPKSLAYWEK     32
                                 :|....|...|     .:......|...|:....|..
PrpF39           51 VNASTEETEMASAVDLPVTLTETEANFPPEYEKFWKTVENNPQDFTGWVY    100

Prp42            33 LLNYIVKASAPICKSTEPQLLKLIRCTYSSMLNEFPYLENYYIDFALLEY     82
                    ||.|:          .:...|...|..:......:||...|:..:|.||.
PrpF39          101 LLQYV----------EQENHLMAARKAFDRFFIHYPYCYGYWKKYADLEK    140

Prp42            83 KLGNVSMSHKIFQRGLQAFNQRSLLLWTSYLKF-----------CNNVIS    121
                    :..|:..|.::::|||||. ..|:.||..|:.|           .||.|.
PrpF39          141 RHDNIKPSDEVYRRGLQAI-PLSVDLWIHYINFLKETLDPGDPETNNTIR    189

Prp42           122 HQKQLFKKYETAEEYVGLHFFSGEFWDLYL----EQISSRCTSSKKYWNV    167
                          ..:|.|....|..|.|...|::|:    ||.:.|..::     :
PrpF39          190 ------GTFEHAVLAAGTDFRSDRLWEMYINWENEQGNLREVTA-----I    228

Prp42           168 LRKILEIPLHSFSKFYALWLQRID-----DIMDLKQLSQLTSKDELLKKL    212
                    ..:||.||...:|..:..:.:.:.     |::..:|..||.      ::|
PrpF39          229 YDRILGIPTQLYSHHFQRFKEHVQNNLPRDLLTGEQFIQLR------REL    272

Prp42           213 KIDINYSGRKGPYLQDAKKKLKKITKEMYMV-----VQYQVLEIYS----    253
                    .....:||..||...|....::.||....::     ::::::||:.
PrpF39          273 ASVNGHSGDDGPPGDDLPSGIEDITDPAKLITEIENMRHRIIEIHQEMFN    322

Prp42           254 ----------IFESKIYINYY-TSPETLVSSDEIETWIKYLDYTITLQTD    292
                              .||..|...|: ..|   :...::..|.:||::.|...|.
PrpF39          323 YNEHEVSKRWTFEEGIKRPYFHVKP---LEKAQLKNWKEYLEFEIENGTH    369

Prp42           293 SLTHLNFQRALLPLAHYDLVWIKYSKWLINSKNDLLGAKNVLLMGLKFSL    342
                    ....:.|:|.::..|.|:..||||:|::.|  :.:.|.::|........|
PrpF39          370 ERVVVLFERCVISCALYEEFWIKYAKYMEN--HSIEGVRHVFSRACTIHL    417

Prp42           343 KKTEIIKLLY--------------SVICKLNEYVL------LRNL-----    367
                    .|..::.:|:              :::....|.||      ||.:
PrpF39          418 PKKPMVHMLWAAFEEQQGNINEARNILKTFEECVLGLAMVRLRRVSLERR    467

Prp42           368 ---LEKIESSYSDNVENVDDFEIFWDYLQFKTFCQNSLYSSRYSDSQSNG    414
                       ||:.|....|.::|.            |:..::|.|:.:
PrpF39          468 HGNLEEAEHLLQDAIKNA------------KSNNESSFYAVK--------    497

Prp42           415 LLNKELFDKVWKRLSCKEK---------KSGQEILLNNLVQFYSKDTVEF    455
                     |.:.|| |:.|.|....|         |..:::.||.|...||.| ::.
PrpF39          498 -LARHLF-KIQKNLPKSRKVLLEAIERDKENTKLYLNLLEMEYSGD-LKQ    544

Prp42           456 VEKNIFQKIIEFGWEYYLQNGMFWNCY-----------CRLIYFDTSRSY    494
                    .|:||.                  ||:           .|:.:......:
PrpF39          545 NEENIL------------------NCFDKAVHGSLPIKMRITFSQRKVEF    576

Prp42           495 LDKRQYIVRKIWPQIDKKFAQSVLPSLTEFCESYFPEEMDTLE---EMFT    541
                    |:.....|.|:....|:.  |::|            :|.|:|:   |..:
PrpF39          577 LEDFGSDVNKLLNAYDEH--QTLL------------KEQDSLKRKAENGS    612

Prp42           542 EEP-----------------------------------------------    544
                    |||
PrpF39          613 EEPEEKKAHTEDTTSSSTQMIDGDLQANQAVYNYSAWYQYNYQNPWNYGQ    662

Prp42           545 -------    544

PrpF39          663 YYPPPPT    669


Alignment length: 757
Identity:    132/757 (17.4%)
Similarity: 236/757 (31.2%)
```

```
Prp39              1 ------------------------MPDETNFTIE-----DIEPRPDALRG     21
                                             :...|:|:.|     ::|..||
PrpF39             1 MQNSHMDEYRNSSNGSTGNSSEVVVEHPTDFSTEIMNVTEMEQSPD----     46

Prp39             22 LDTQFLQDNTALVQAYRGLD-------------------W-------SDI     45
                      |:..:..:|..:....:|                   |       .|.
PrpF39            47 -DSPNVNASTEETEMASAVDLPVTLTETEANFPPEYEKFWKTVENNPQDF     95

Prp39             46 SSLTQMVDVIEQTVVKYGNPNDSIKLALETILWQILRKYPLLFGFWKRFA     95
                     :....::..:||         ::..:|......:....||..:|:||::|
PrpF39            96 TGWVYLLQYVEQ---------ENHLMAARKAFDRFFIHYPYCYGYWKKYA    136

Prp39             96 TIEYQLFGLKKSIAVLATSVKWFPTSLELWCDYLN----VLCVNNPNETD    141
                     .:|.:...:|.|..|....::..|.|::||..|:|    .|...:|...:
PrpF39           137 DLEKRHDNIKPSDEVYRRGLQAIPLSVDLWIHYINFLKETLDPGDPETNN    186

Prp39            142 FIRNNFEIAKDLIGKQFLSHPFWDKFIEFEVGQKNWHNVQRIYEYIIEVP    191
                     .||..||.|....|..|.|...|:.:|.:|..|.|...|..||:.|:.:|
PrpF39           187 TIRGTFEHAVLAAGTDFRSDRLWEMYINWENEQGNLREVTAIYDRILGIP    236

Prp39            192 LHQYARFFTSYKKFLNE---KNLKTTRNIDIVLRKTQTTVN---------    229
                     ...|:..|..:|:.:..   ::|.|.... |.||:...:||
PrpF39           237 TQLYSHHFQRFKEHVQNNLPRDLLTGEQF-IQLRRELASVNGHSGDDGPP    285

Prp39            230 -------------------EI-------------------------WQFE    235
                                        ||                         |.||
PrpF39           286 GDDLPSGIEDITDPAKLITEIENMRHRIIEIHQEMFNYNEHEVSKRWTFE    335

Prp39            236 SKIKQPFFNLGQVLNDDLENWSRYLKFVTDPSKSLDKEFVMSVFDRCLIP    285
                     ..||:|:|::..:....|:||..||:|..:..   ..|.|:.:|:||:|.
PrpF39           336 EGIKRPYFHVKPLEKAQLKNWKEYLEFEIENG---THERVVVLFERCVIS    382

Prp39            286 CLYHENTWMMYIKWLTKKNISDEVVVDIYQKANTF-LPLDFKTLRYDFLR    334
                     |..:|..|:.|.|::  :|.|.|.|..::.:|.|. ||      :...:.
PrpF39           383 CALYEEFWIKYAKYM--ENHSIEGVRHVFSRACTIHLP------KKPMVH    424

Prp39            335 FLKRKYRSNNTLFNNIFNETVSRYLKIWPNDILLMTEYLCMLKRHSFK--    382
                     .|...:.......|...|     .||.:...:|.:.  :..|:|.|.:
PrpF39           425 MLWAAFEEQQGNINEARN-----ILKTFEECVLGLA--MVRLRRVSLERR    467

Prp39            383 -NSLDQSPKEILEKQTSFTKILETSITNYINNQIDAKVHLQTLINDKNLS    431
                      .:|:::           ..:|:.:|.|..:|            |:.  |
PrpF39           468 HGNLEEA-----------EHLLQDAIKNAKSN------------NES--S    492

Prp39            432 IVVVELIKTTWLVLKNNMQTRK---------------YFNLY--------    458
                     ...|:|.:..:.:.||..::||               |.||.
PrpF39           493 FYAVKLARHLFKIQKNLPKSRKVLLEAIERDKENTKLYLNLLEMEYSGDL    542

Prp39            459 ---QKNIL------IKNSVPFWLTYYKFEKSNVNFTKLN-KFIRELGVEI    498
                        ::|||      :..|:|.        |..:.|::.. :|:.:.|.::
PrpF39           543 KQNEENILNCFDKAVHGSLPI--------KMRITFSQRKVEFLEDFGSDV    584

Prp39            499 YLPTTVMNDILTDYKTFYLTHSNIVTYESSIIDSNTFDPILYPELKMSNP    548
                            |.:|..|.    .|..::..:.|:            :.|..|.
PrpF39           585 -------NKLLNAYD----EHQTLLKEQDSL------------KRKAENG    611

Prp39            549 KYDPVLNTTANVDWHKKTEWKEAGHIGITTERPQISNSIIECNS------    592
                     ..:|              |.|:|.....|:...|:.:..::.|.
PrpF39           612 SEEP--------------EEKKAHTEDTTSSSTQMIDGDLQANQAVYNYS    647

Prp39            593 -------------GTLIQKPISLPNFRNLEKINQVKINDLYTEEFLKEGK    629
                                  |.....|.:
PrpF39           648 AWYQYNYQNPWNYGQYYPPPPT----------------------------    669
```

Alignment length: 800
Identity: 144/800 (18.0%)
Similarity: 260/800 (32.5%)

```
Prp40              1 --------------------------------------------------      0

PRP40FA            1 MRPGTGAERGGLMVSEMESHPPSQGPGDGERRLSGSSLCSGSWVSADGFL     50

Prp40              1 --------------------------------------------------      0

PRP40FA           51 RRRPSMGHPGMHYAPMGMHPMGQRANMPPVPHGMMPQMMPPMGGPPMGQM    100

Prp40              1 ------------------------------------------MSIWKEAK      8
                                                               .|:|.|.|
PRP40FA          101 PGMMSSVMPGMMMSHMSQASMQPALPPGVNSMDVAAGTASGAKSMWTEHK    150

Prp40              9 DASGRIYYYNTLTKKSTWEKPKELISQEELLLRENGWKAAKTADGKVYYY     58
                     ...||.|||||.||:||||||.:|.:..|.||.:..||..|:..||.|||
PRP40FA          151 SPDGRTYYYNTETKQSTWEKPDDLKTPAEQLLSKCPWKEYKSDSGKPYYY    200

Prp40             59 NPTTRETSWTIP-------------------------AFEKKVEPIAEQK     83
                     |..|:|:.|..|                         |..|..|...:::
PRP40FA          201 NSQTKESRWAKPKELEDLEGYQNTIVAGSLITKSNLHAMIKAEESSKQEE    250

Prp40             84 HDTVSHAQV-----------------------------------------     92
                     ..|.|.|.|
PRP40FA          251 CTTTSTAPVPTTEIPTTMSTMAAAEAAAAVVAAAAAAAAAAAAANANAST    300

Prp40             93 ---------------------------NGNRIALT---------------    100
                                                |.|.:.::
PRP40FA          301 SASNTVSGTVPVVPEPEVTSIVATVVDNENTVTISTEEQAQLTSTPAIQD    350

Prp40            101 --------AGEKQEPGRTI-------NEEESQYANNSKLLNVRRRTKEEA    135
                             .||:.....|:       .|||||.|..:...|    |||||
PRP40FA          351 QSVEVSSNTGEETSKQETVADFTPKKEEEESQPAKKTYTWN----TKEEA    396

Prp40            136 EKEFITMLKENQVDSTWSFSRIISELGTRDPRYWMVDDDPLW--------    177
                     ::.|..:|||.:|.|..|:.:.:.          |:.:||.:
PRP40FA          397 KQAFKELLKEKRVPSNASWEQAMK----------MIINDPRYSALAKLSE    436

Prp40            178 KKEMFEKYLSNRSADQLLKEHNE--TSKFKEA---FQKMLQNNSHIKYYT    222
                     ||:.|..|     ..|..||..|  .||:|||   ||:.|:|:.:...|
PRP40FA          437 KKQAFNAY-----KVQTEKEEKEEARSKYKEAKESFQRFLENHEKMTSTT    481

Prp40            223 RWPTAKRLIADEPIYKHSVVNEKTKRQTFQDYIDTLIDTQKESKKKLKTQ    272
                     |:..|:::..:..::  :.::|:.:.:.::|.:..|...:||..|:|:.:
PRP40FA          482 RYKKAEQMFGEMEVW--NAISERDRLEIYEDVLFFLSKKEKEQAKQLRKR    529

Prp40            273 ALKELREYLNGIITTSSSETFITWQQLLNHYVFDKSKRYMANRHFKVLTH    322
                     ..:.|:..|:.:...:.|.|:...||    |:.| :..:.:...:.:..
PRP40FA          530 NWEALKNILDNMANVTYSTTWSEAQQ----YLMD-NPTFAEDEELQNMDK    574

Prp40            323 EDVLNEYLKIVNTIENDLQNKLNELRLRNYTRDRIARDNFKSLLREVPI-    371
                     ||.|..:.:.:.:|.:.:.:.:..||...|.|..|::|:..|.|:..
PRP40FA          575 EDALICFEEHIRALEKEEEEEKQKSLLRERRRQRKNRESFQIFLDELHEH    624

Prp40            372 -KIKANTRWSDIYPHIKSDPRFLHMLGRNGSSCLDLFLDFVDEQRMYIFA    420
                      ::.:.:.|.::||.|.||.||.:|||:.||:.||||..:|::.:.....
PRP40FA          625 GQLHSMSSWMELYPTISSDIRFTNMLGQPGSTALDLFKFYVEDLKARYHD    674

Prp40            421 QRSIAQQTLIDQNF--EWNDADSDEITKQNIEKVLENDRKFDKVDKEDIS    468
                     ::.|.:..|.|:.|  |.|....|.:.      ::.:.::...:|..:|.
PRP40FA          675 EKKIIKDILKDKGFVVEVNTTFEDFVA------IISSTKRSTTLDAGNIK    718

Prp40            469 LIVDGLIKQRNEKIQQKLQNERRILEQKKHYFWLLLQRTYTKTGKPKPST    518
                     |..:.|::...:.::.:.|.|.:::|:..|     ::..|...| |..
PRP40FA          719 LAFNSLLEKAEARERERERKEEARKMKRKESAF-----KSMLKQAAP-PIE    762

Prp40            519 WDLASKELGESLEYKALGDE---DNIRRQIFEDF----------------    549
                     .|...:::.|....:...::   ::.|::||:||
PRP40FA          763 LDAVWEDIRERFVKEPAFEDITLESERKRIFKDFMHVLEHECQHHHSKNK    812

Prp40            550 -----------------------------------KPESSAPTAESATANL    565
                                                        :.||.:.:..|::|..
PRP40FA          813 KHSKKSKKHHRKRSRSRSGSDSDDDDSHSKKKRQRSESRSASEHSSSAES    862

Prp40            566 TLT----------ASKKRHLTPAVELDY----------------------    583
                     ..:          :.|:||.:.:.|.|.
PRP40FA          863 ERSYKKSKKHKKKSKKRRHKSDSPESDAEREKDKKEKDRESEKDRTRQRS    912

Prp40            584 ---------------------------------------------    583

PRP40FA          913 ESKHKSPKKKTGKDSGNWDTSGSELSEGELEKRRRTLLEQLDDDQ    957

                 Alignment length: 995
                 Identity:    169/995 (17.0%)
                 Similarity: 307/995 (45.2%)
```

```
Nam8         1 MSYKQTTYYPSRGNLVRNDSSPYTNTISSETNNSSTSVLSLQGASNVSLG     50
                                                              ...
TIA1         1 -----------------------------------------------MED      3

Nam8        51 TTGNQLYMGDLDPTWDKNTVRQIWASLGEANINVRMMWNNTLNNGSRSSM    100
               .....||:|:|....:..:..|:::.:|... |.:|:            |
TIA1         4 EMPKTLYVGNLSRDVTEALILQLFSQIGPCK-NCKMI------------M     40

Nam8       101 GPKNNQGYCFVDFPSSTHAANAL-LKNGMLIPNFPNKKLKLNWATSSYS-    148
               ....|..||||:|....|||.|| ..||..|   ..|::|:||||:..|
TIA1        41 DTAGNDPYCFVEFHEHRHAAAALAAMNGRKI---MGKEVKVNWATTPSSQ     87

Nam8       149 ----NSNNSLNNVKSGNNCSIFVGDLAPNVTESQLFELFINRYASTSHAK    194
                   :|:..::..:|.::..:|||||:|.:|...:...|. .:...|.|:
TIA1        88 KKDTSSSTVVSTQRSQDHFHVFVGDLSPEITTEDIKAAFA-PFGRISDAR    136

Nam8       195 IVHDQVTGMSKGYGFVKFTNSDEQQLALSEMQGVFLNGRAIKVGPTSGQQ    244
               :|.|..||.|||||||.|.|..:.:.|:.:|.|.:|.||.|:....:  :
TIA1       137 VVKDMATGKSKGYGFVSFFNKWDAENAIQQMGGQWLGGRQIRTNWAT--R    184

Nam8       245 QHVSGNNDYNRSSSSLNNENVDSRFLSKGQSFLSNGNNNMGFKRNHMSQF    294
               :..:..:.|..::..|:.:.|
TIA1       185 KPPAPKSTYESNTKQLSYDEV-----------------------------    205

Nam8       295 IYPVQQQPSLNHFTDPNNTTVFIGGLSSLVTEDELRAYFQPFGTIVYVKI    344
                  |.|       :.|:|.||:.||::|.:||..:|..|.|||.|:.:::
TIA1       206 ---VNQ-------SSPSNCTVYCGGVTSGLTEQLMRQTFSPFGQIMEIRV    245

Nam8       345 PVGKCCGFVQYVDRLSAEAAIAGMQGFPIANSRVRLSWGRSAKQ--TALL    392
               ...|...||::....||..||..:.|..|....|:..||:....  ..:.
TIA1       246 FPDKGYSFVRFNSHESAAHAIVSVNGTTIEGHVVKCYWGKETLDMINPVQ    295

Nam8       393 QQAML-------------SNSLQVQQQQP-GLQQPNYGY---------IP    419
               ||..:             .|:.|:.|..| |.|.|.||.         ..
TIA1       296 QQNQIGYPQPYGQWGQWYGNAQQIGQYMPNGWQVPAYGMYGQAWNQQGFN    345

Nam8       420 SSTCEAP-------VLPDNNVSSTMLP----GCQILNYSNPYANANGLGS    458
               .:...||       |.|...:.:.|||    |.::..|...
TIA1       346 QTQSSAPWMGPNYGVQPPQGQNGSMLPNQPSGYRVAGYETQ---------    386

Nam8       459 NNFSFYSNNNATNTQATSLLADTSSMDLSGTGGQQVIMQGSEAVVNSTNA    508

TIA1       387 --------------------------------------------------    386

Nam8       509 MLNRLEQGSNGFMFA    523

TIA1       387 ---------------    386
```

Alignment length: 565
Identity: 114/565 (20.2%)
Similarity: 183/565 (32.4%)

```
Snu71              1 --------------------------------------------------     0

RBM25              1 MSFPPHLNRPPMGIPALPPGIPPPQFPGFPPPVPPGTPMIPVPMSIMAPA    50

Snu71              1 --------------------------------------------------     0

RBM25             51 PTVLVPTVSMVGKHLGARKDHPGLKAKENDENCGPTTTVFVGNISEKASD   100

Snu71              1 --MRDIVFVSPQLYLSSQEGWKSDSAKSGFIPI-----------------    31
                       :|.:: ....|.||    ||.....||.:..
RBM25            101 MLIRQLL-AKCGLVLS----WKRVQGASGKLQAFGFCEYKEPESTLRALR   145

Snu71             32 LKNDLQRFQDSLKHIVDARNSLSETLLNSNDDGSIHNSDQNTGLNKDKEA    81
                     |.:|||..:..|...|||:.........:....|..|:...|..|.|:||
RBM25            146 LLHDLQIGEKKLLVKVDAKTKAQLDEWKAKKKASNGNARPETVTNDDEEA   195

Snu71             82 SIADNNSANKCATSSSRYQELKQFLPISLDQQIHTVSLQGVSSSFSRGQI   131
                       .|..:..:                   ||.|             :|.|
RBM25            196 --LDEETKRR-------------------DQMI-------------KGAI   211

Snu71            132 ESLLDHC---LNLALTETQSNSALKVEAWSSFSSFLDTQDIFIRF-----   173
                     |.|:...   ||....|:.|:...|.:.        ..:|||.||
RBM25            212 EVLIREYSSELNAPSQESDSHPRKKKKE--------KKEDIFRRFPVAPL   253

Snu71            174 ------SKVDEDEAFVNTLNYCKALFAFIRKLHEDFKIELHLDLNTKEY-   216
                           :|.|     :|.:           ::.||.:     ||.::|.
RBM25            254 IPYPLITKED-----INAI-----------EMEEDKR-----DLISREIS   282

Snu71            217 -VEDRTGTIPSVKPEKASEFYSVFKNIEDQTDERNSKKEQLD--------   257
                      ..|....:...|.:|..|...:.|...::..||..::|:.:
RBM25            283 KFRDTHKKLEEEKGKKEKERQEIEKERRERERERERERRERERERERERE   332

Snu71            258 -------------------DSSTQYKVDTNTLSDLPSD---ALDQLCKDI   285
                                        |.:.:...|.:...|...|   :.|: .||.
RBM25            333 REREKEKERERERERDRDRDRTKERDRDRDRERDRDRDRERSSDR-NKDR   381

Snu71            286 IEFRTKVVSIEKEKKMKSTYEESRRQRHQMQKVFDQIRKNHSGAKGSANT   335
                     ...|.|....|:|::.:...|..|.:..:.::..::.|:.
RBM25            382 SRSREKSRDRERERERERERERERERERERERERERERER----------   421

Snu71            336 EEEDTNMEDEDEEDDTEDDLALEKRKEERDLEESNRRYEDMLHQ------   379
                     |.|....:..|.|:|.||  |.|:||.||.|.|....|::.|..
RBM25            422 EREREKDKKRDREEDEED--AYERRKLERKLREKEAAYQERLKNWEIRER   469

Snu71            380 ------------------------------------------------LH   381
                                                                     |.
RBM25            470 KKTREYEKEAEREEERRREMAKEAKRLKEFLEDYDDDRDDPKYYRGSALQ   519

Snu71            382 SNTEPKIKSIRADIMSAENYEEHLEKNRSLYL--------KELLHLANDV   423
                     .....:.|.:.||....:..:|.||:.|...|        .||..:..:.
RBM25            520 KRLRDREKEMEADERDRKREKEELEEIRQRLLAEGHPDPDAELQRMEQEA   569

Snu71            424 HYDHHRSFK-------EQEERRDEEDRAKNGNAKE------------LAP   454
                     ........|       |:||::::|::.:....:|            |.|
RBM25            570 ERRRQPQIKQEPESEEEEEEKQEKEEKREEPMEEEEEPEQKPCLKPTLRP   619

Snu71            455 IQLSDGKAISAGKAAAIT----LPEGTVKSENYNADKNVSESSEH-----   495
                     |..:...:.::|.|...|    .|.|.:.....:.|:...|  ||
RBM25            620 ISSAPSVSSASGNATPNTPGDESPCGIIIPHENSPDQQQPE--EHRPKIG   667

Snu71            496 ----------------VKIKFDFKKAIDHSVESSSEDE------------   517
                                     ||.|   |..:| ||.:..|||
RBM25            668 LSLKLGASNSPGQPNSVKRK---KLPVD-SVFNKFEDEDSDDVPRKRKLV   713

Snu71            518 --GYRESELPPTKPSERSAAE--------DRLPFTADEL-------NIRL   550
                       .|.|.:...||.:..:..:        :::|....||       :|..
RBM25            714 PLDYGEDDKNATKGTVNTEEKRKHIKSLIEKIPTAKPELFAYPLDWSIVD   763

Snu71            551 TNLKESR---YVDELVREFLGVYEDELVEYILENIRVNQSKQALLNELRE   597
                     :.|.|.|   ::::.:.|::|..|..||:::...:..:.|.|::|:::..
RBM25            764 SILMERRIRPWINKKIIEYIGEEEATLVDFVCSKVMAHSSPQSILDDVAM   813

Snu71            598 TFDEDGETIADRLW-----SRKEFRLGT--    620
                     ..||:.|....::|     ..:..::|.
RBM25            814 VLDEEAEVFIVKMWRLLIYETEAKKIGLVK    843
```

```
Alignment length: 930
Identity:    151/930 (16.2%)
Similarity: 397/930 (42.7%)
```

```
Luc7                 1 MSTMSTPAAEQRKLVEQLMG--RDFSFRHNRYSHQKRDLGLHDPKICKSY     48
                           .:..|:.|.|::||||  ||......|       :...|.::|||:
Luc7L                1 ----MSAQAQMRALLDQLMGTARDGDETRQR-------VKFTDDRVCKSH     39

Luc7                49 LVGECPYDLFQGTKQSLGKCPQMHLTKHKIQYEREVKQGKTFPEFEREYL     98
                       |:..||:|:..||:..||:|.::|....:..||...|:...|  ||.:.:
Luc7L               40 LLDCCPHDILAGTRMDLGECTKIHDLALRADYEIASKERDLF--FELDAM     87

Luc7                99 AILSRFVNECNGQISVALQNLKHTAEE--------RMKIQQVTEELDVLD    140
                       ..|..|:.||:.:..:|.:.|..|.||        ..|:.::.||:..|.
Luc7L               88 DHLESFIAECDRRTELAKKRLAETQEEISAEVSAKAEKVHELNEEIGKLL    137

Luc7               141 VRIGLMGQEIDSLIRADEVSMGMLQSVKLQELISKRKEVAKRVRNITENV    190
                       .:...:|.|.:    .|| |..:|..|  :::.:|:||..:..|| :...
Luc7L              138 AKAEQLGAEGN----VDE-SQKILMEV--EKVRAKKKEAEEEYRN-SMPA    179

Luc7               191 GQSAQQKLQVCEVCGAYLSRLDTDRRLADHFLGKIHLGYVKMREDYDRLM    240
                       ....||||:|||||.|||...|.||||||||.||:|||::::||..|:|.
Luc7L              180 SSFQQQKLRVCEVCSAYLGLHDNDRRLADHFGGKLHLGFIQIREKLDQLR    229

Luc7               241 KNNRTTNASKTATTLPGRRFV-----------------------------    261
                       |........:....|..|...
Luc7L              230 KTVAEKQEKRNQDRLRRREEREREERLSRRSGSRTDRRRSRSRDRRRRR    279

Luc7               262 --------------------------------------------------    261

Luc7L              280 SRSTSRERRKLSRSRSRDRHRRHRSRSRSHSRGHRRASRDRSAKYKFSRE    329

Luc7               262 ------------------------------------------    261

Luc7L              330 RASREESWESGRSERGPPDWRLESSNGKMASRRSEEKEAGEI    371
```

```
Alignment length: 392
Identity:    88/392 (22.4%)
Similarity: 135/392 (34.4%)
```

Supplementary Fig. 1. Sequence alignment between yeast U1 auxiliary proteins and their human homologs show significant sequence similarity between the homologs. Alignment was performed using EMBOSS Needle (www.ebi.ac.uk/Tools/psa/emboss_needle/).

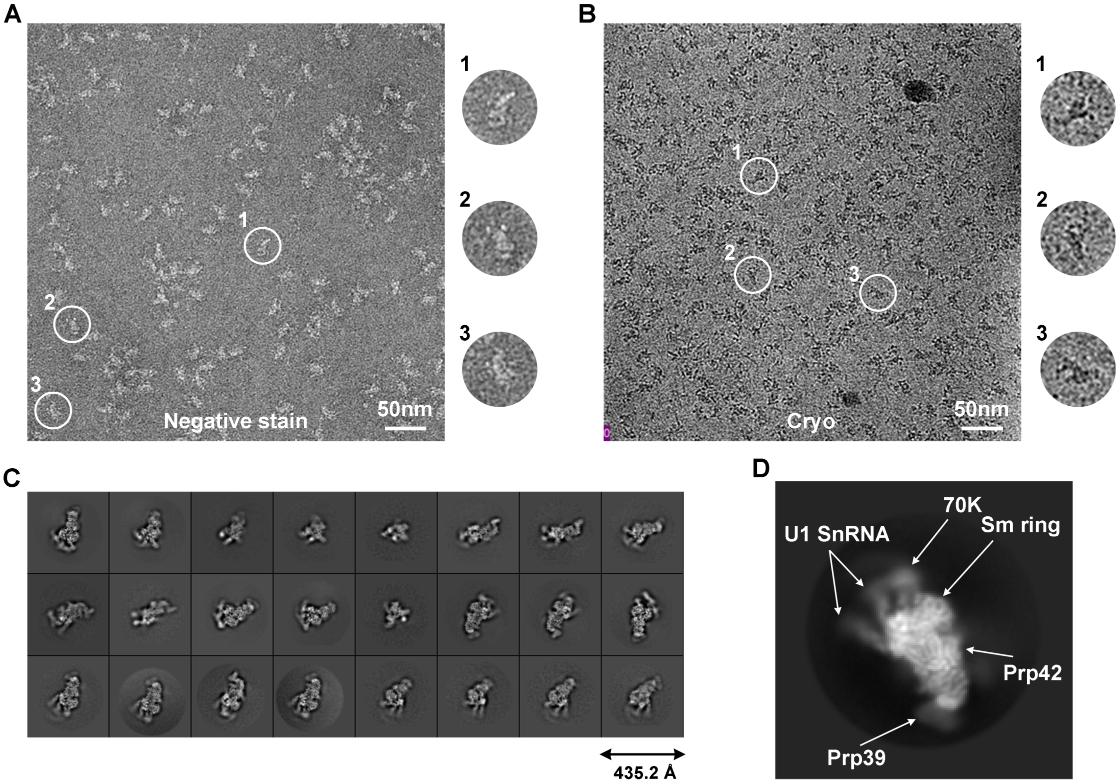

Supplementary Fig. 2. EM images of yeast U1 snRNP particles. (A) A negative-stain EM micrograph of yeast U1 snRNP. (B) A drift-corrected cryoEM micrograph of yeast U1 snRNP. (C) Representative 2D class averages of yeast U1 snRNP obtained in RELION. (D) A highly abundant 2D class average image showing major domains of yeast U1 snRNP.

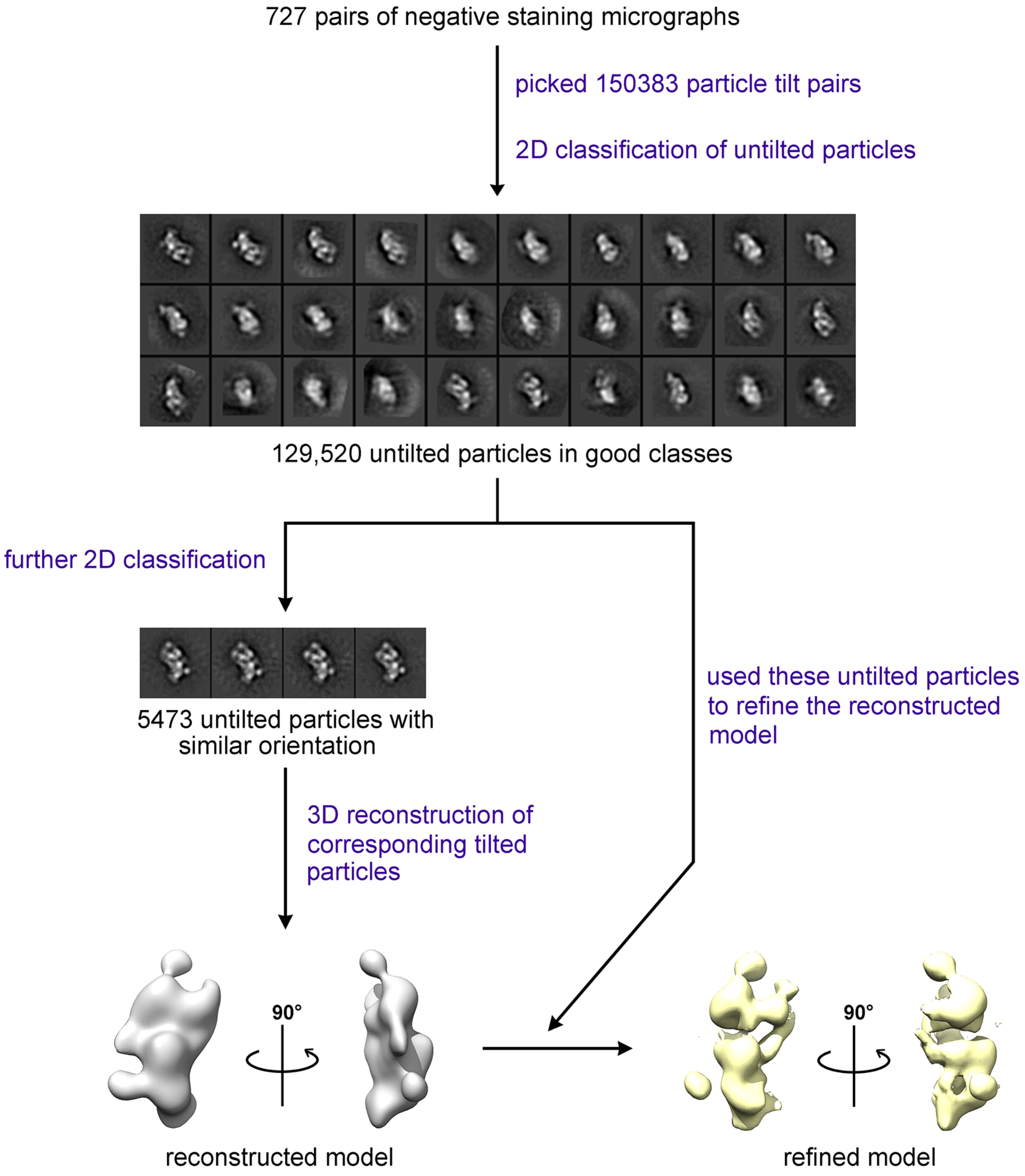

Supplementary Fig. 3. An initial model was generated from negative-stain images using random conical tilt (RCT). Please refer to Materials and Methods for more detail.

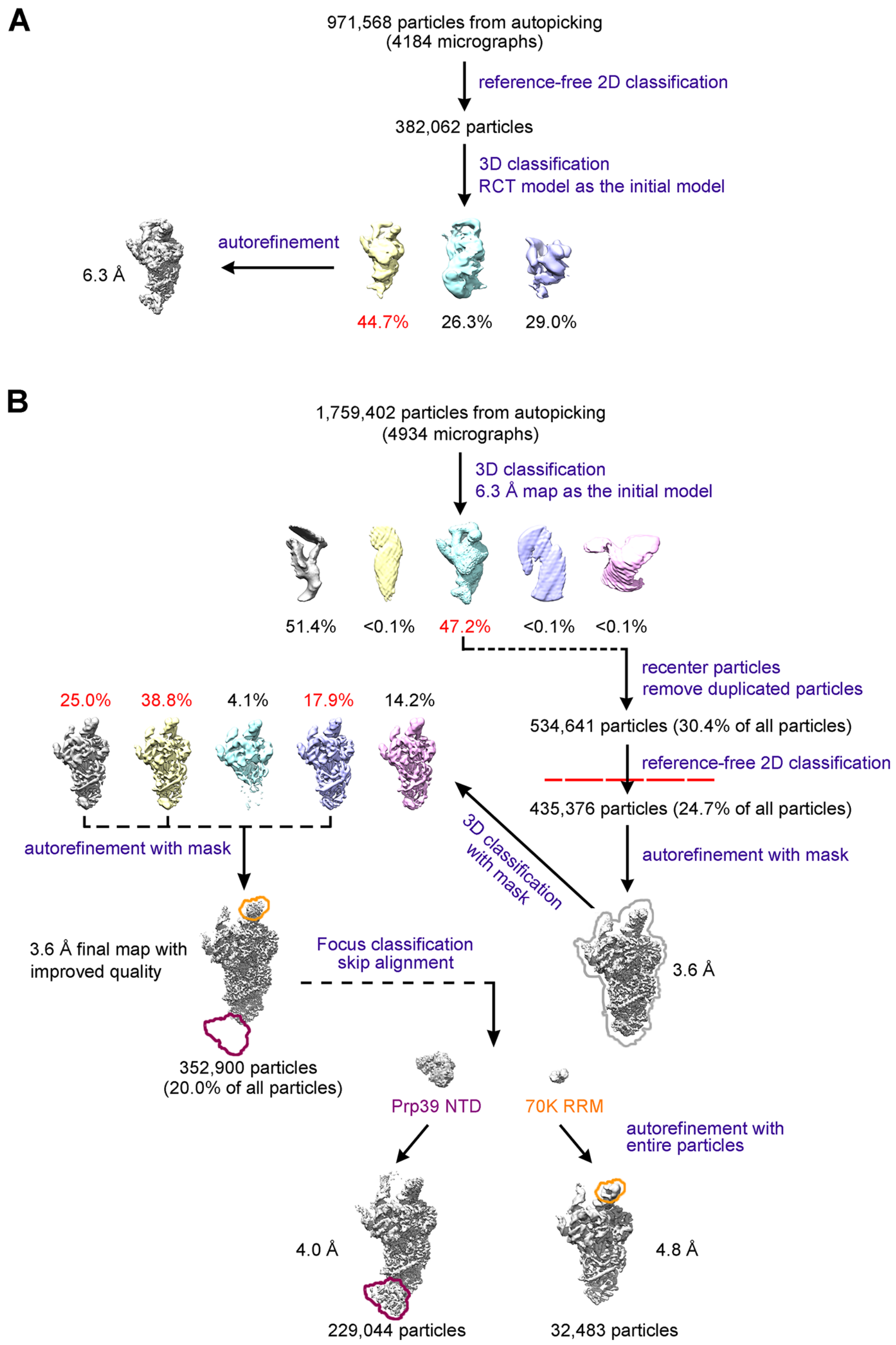

Supplementary Fig. 4. The cryoEM data processing workflow. For data processing above the red dash line, the particle images were binned to a pixel size of 2.72 Å. The rest of data processing was performed with a pixel size of 1.36 Å. Please refer to Materials and Methods for more details.

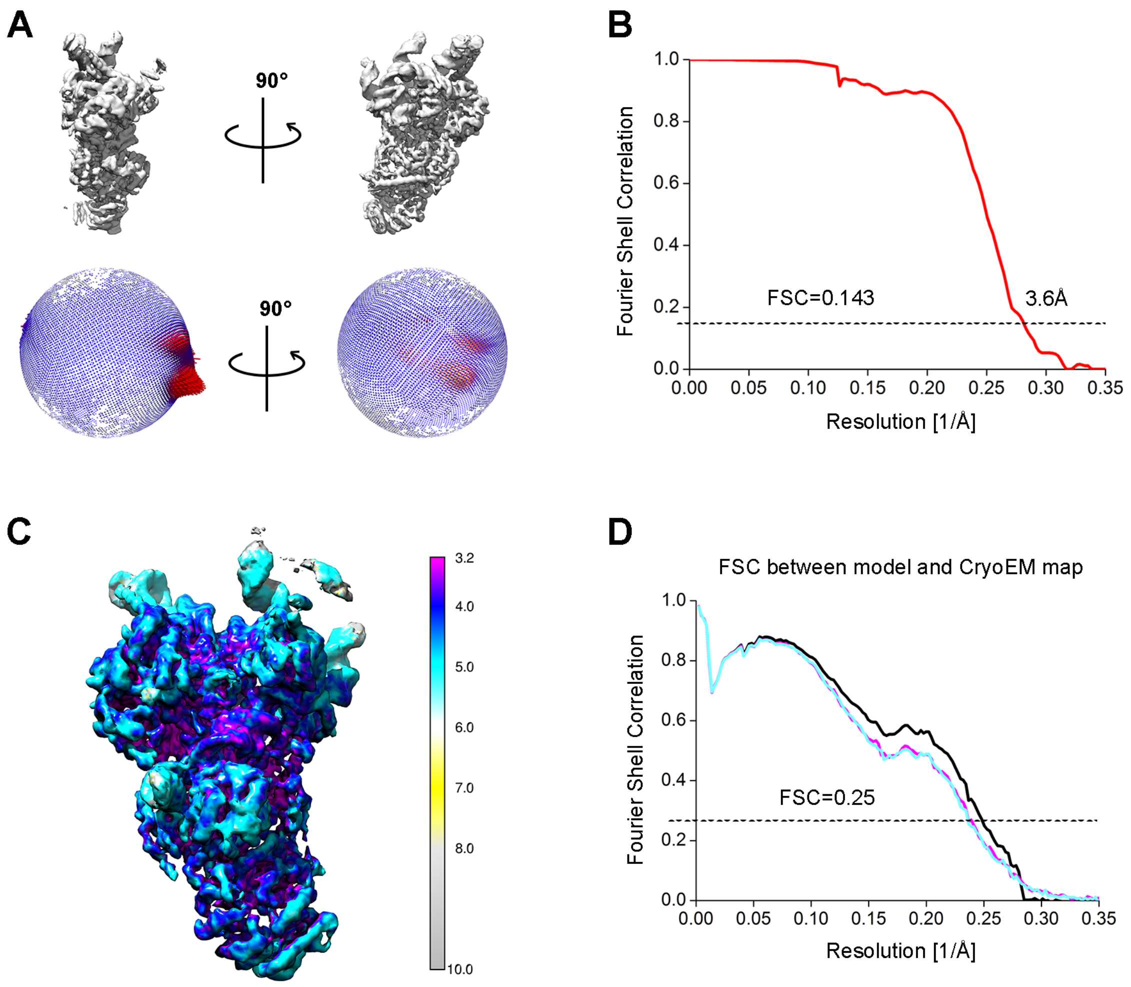

Supplementary Fig. 5. Angular distribution and FSC curves for yeast U1 snRNP. (A) Angular distribution for the final 3.6 Å map of yeast U1 snRNP. (B) FSC curves and calculated resolution for the final reconstruction of yeast U1 snRNP. (C) Resmap local resolution estimation for the core map. (D) FSC curves of the final refined model versus the overall 3.6 Å map (black) and two independent maps used for gold-standard FSC (purple and cyan).

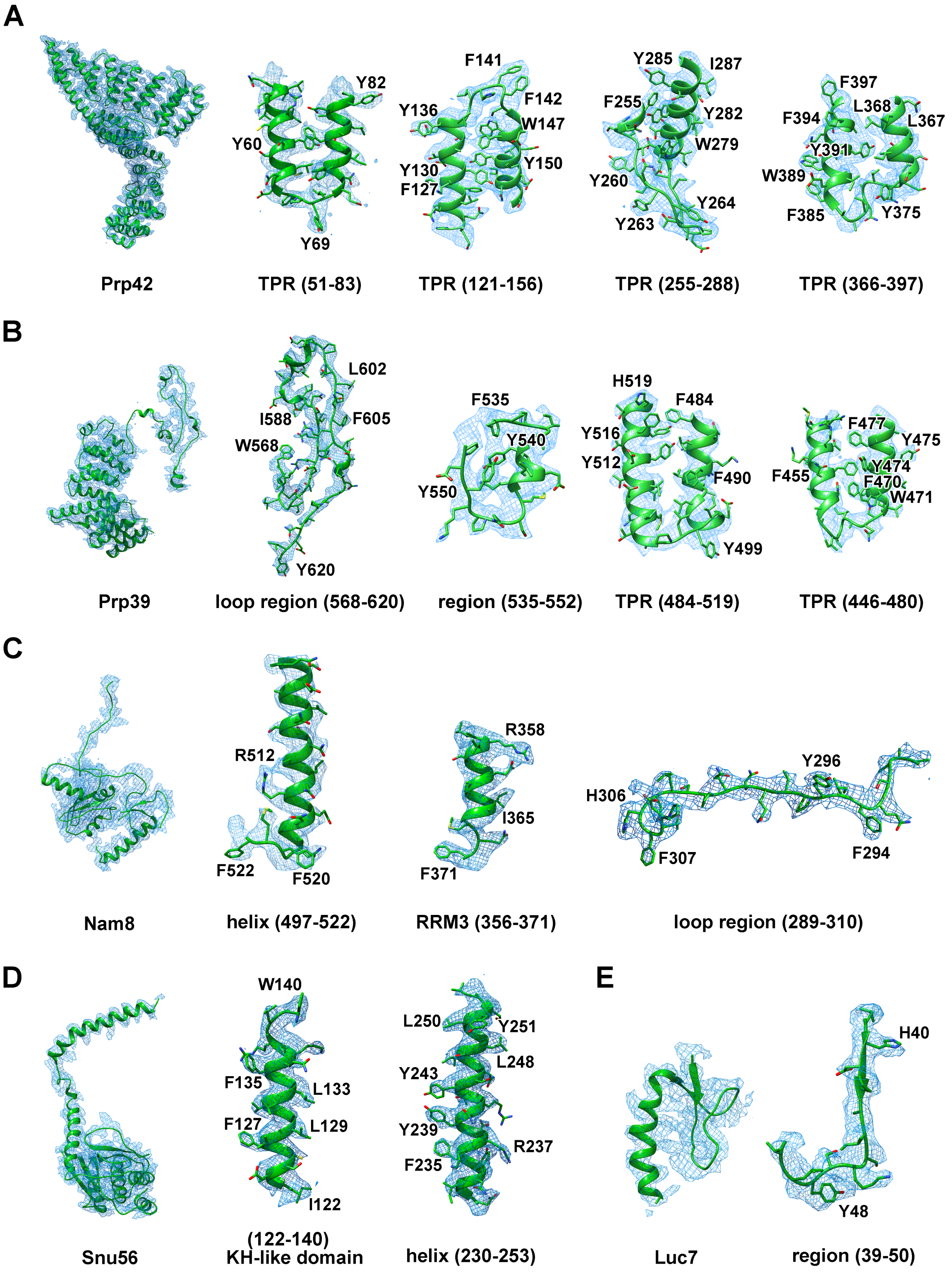

Supplementary Fig. 6. Representative EM density maps of yeast auxiliary U1 snRNP proteins. The EM density maps are shown for Prp42 and its TPR motifs (A); Prp39 and its C-terminal loop region as well as TPR motifs (B); Nam8, the C-terminal helix, representative RRM3 domain secondary structural elements, and the inter-RRM loop (C); Snu56, representative KH-like domain secondary structural elements, and the flanking long helix (D); Luc7 and its N-terminal segment (E).

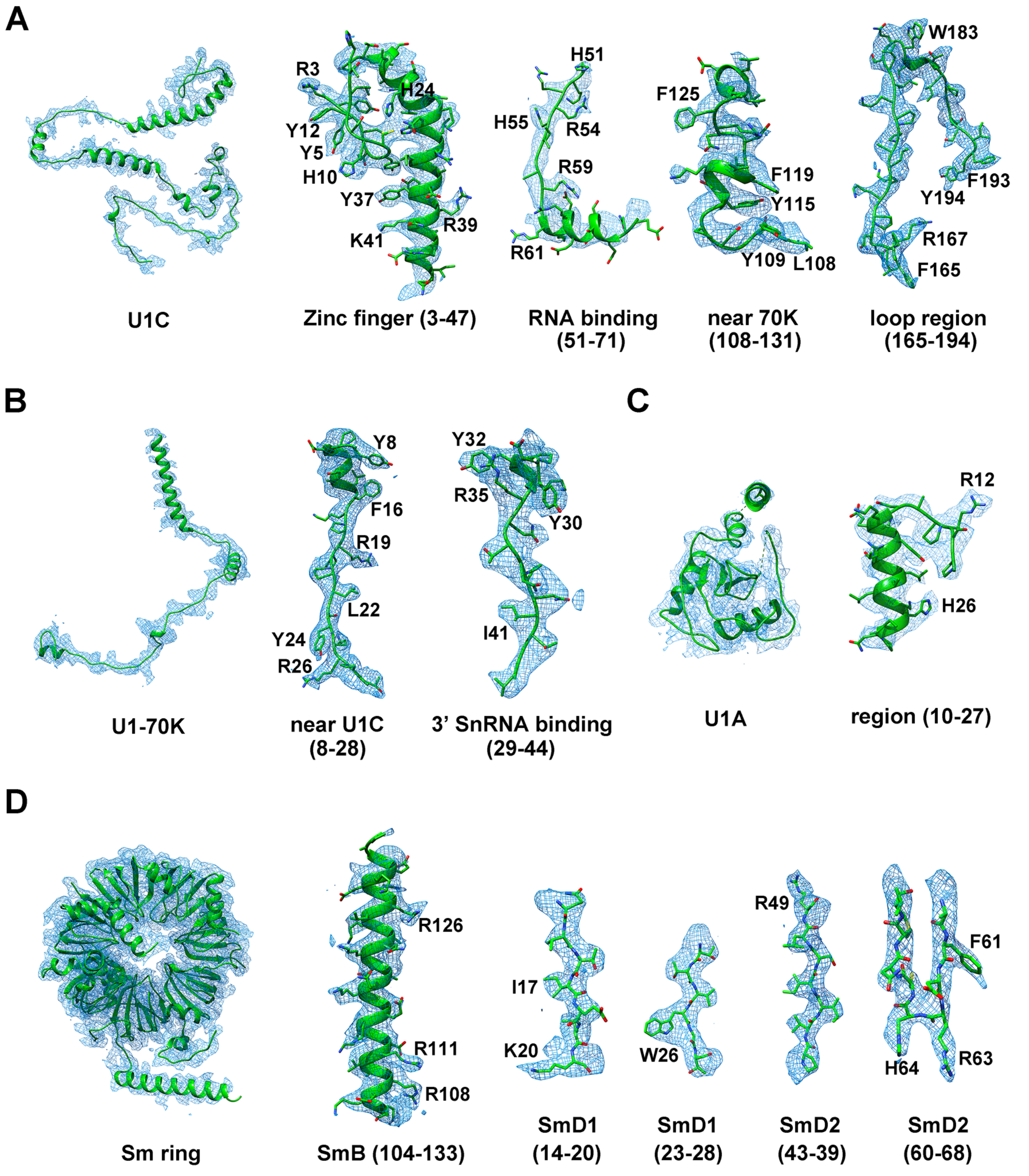

Supplementary Fig. 7. EM density maps of yeast core U1 snRNP proteins. The EM density maps are shown for U1C and segments of the molecule (A); U1-70K and various segments (B); U1A and its N-terminal segment (C); Sm ring and representative secondary structural elements (D).

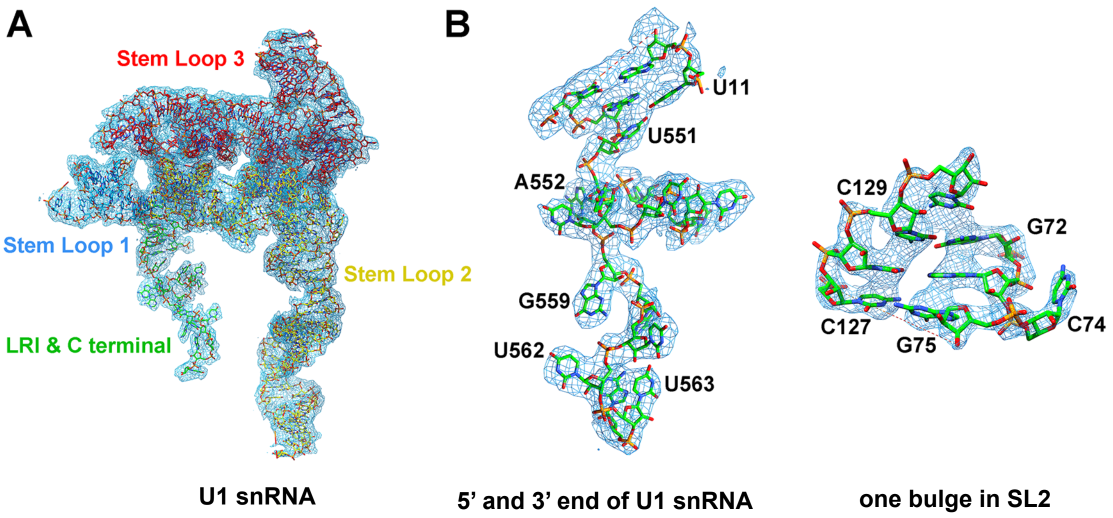

Supplementary Fig. 8. EM density maps of yeast U1 snRNA. (A) Overall density map of U1 snRNA. (B) EM density maps for the 5' region and 3' Sm-binding region. (C) EM density for a RNA bulge in Stem/Loop (SL) 2.

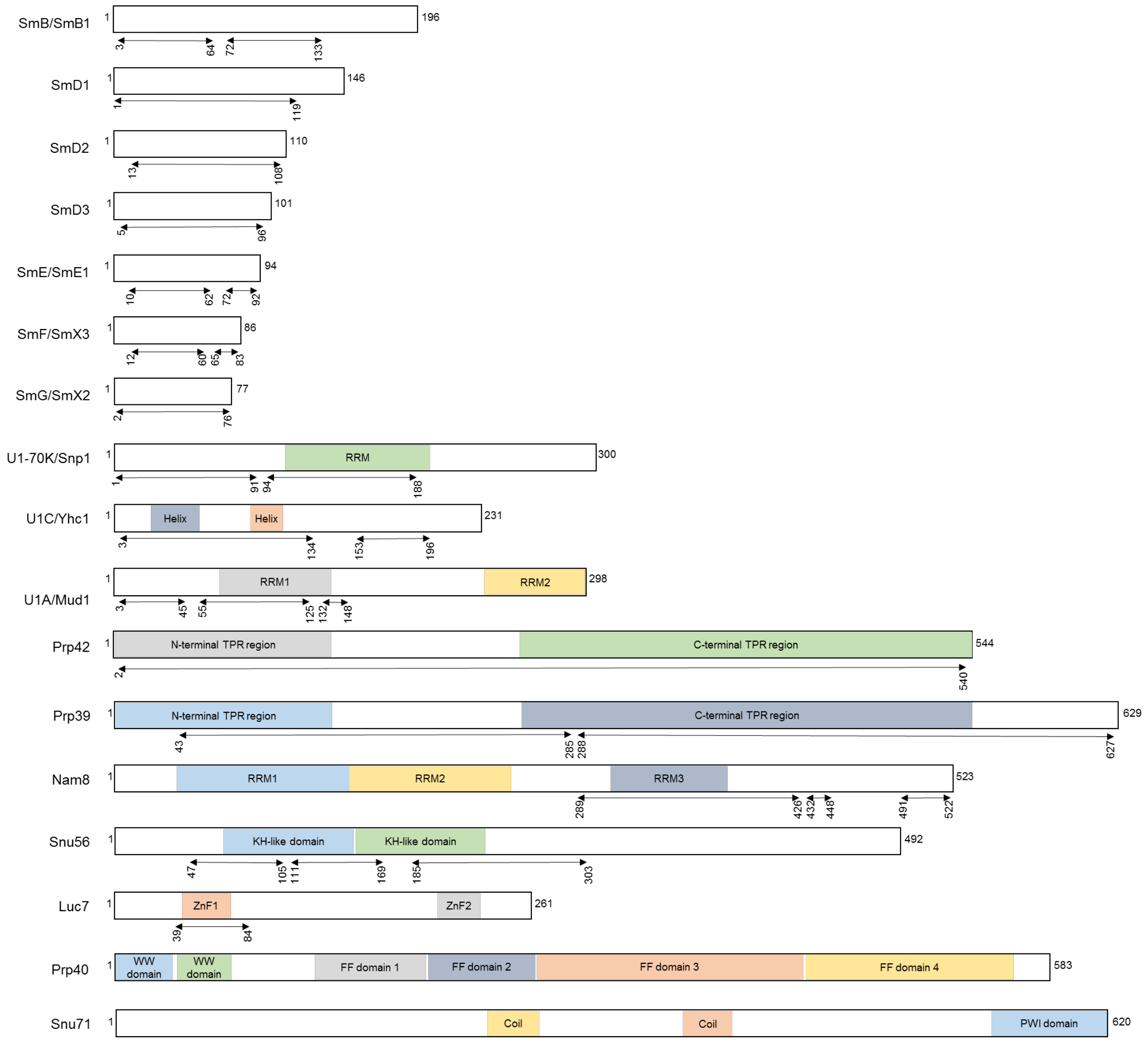

Supplementary Fig. 9. Schematic diagrams of the protein components of the yeast U1 snRNP. Regions where models have been built into the structure are shown in double arrows.

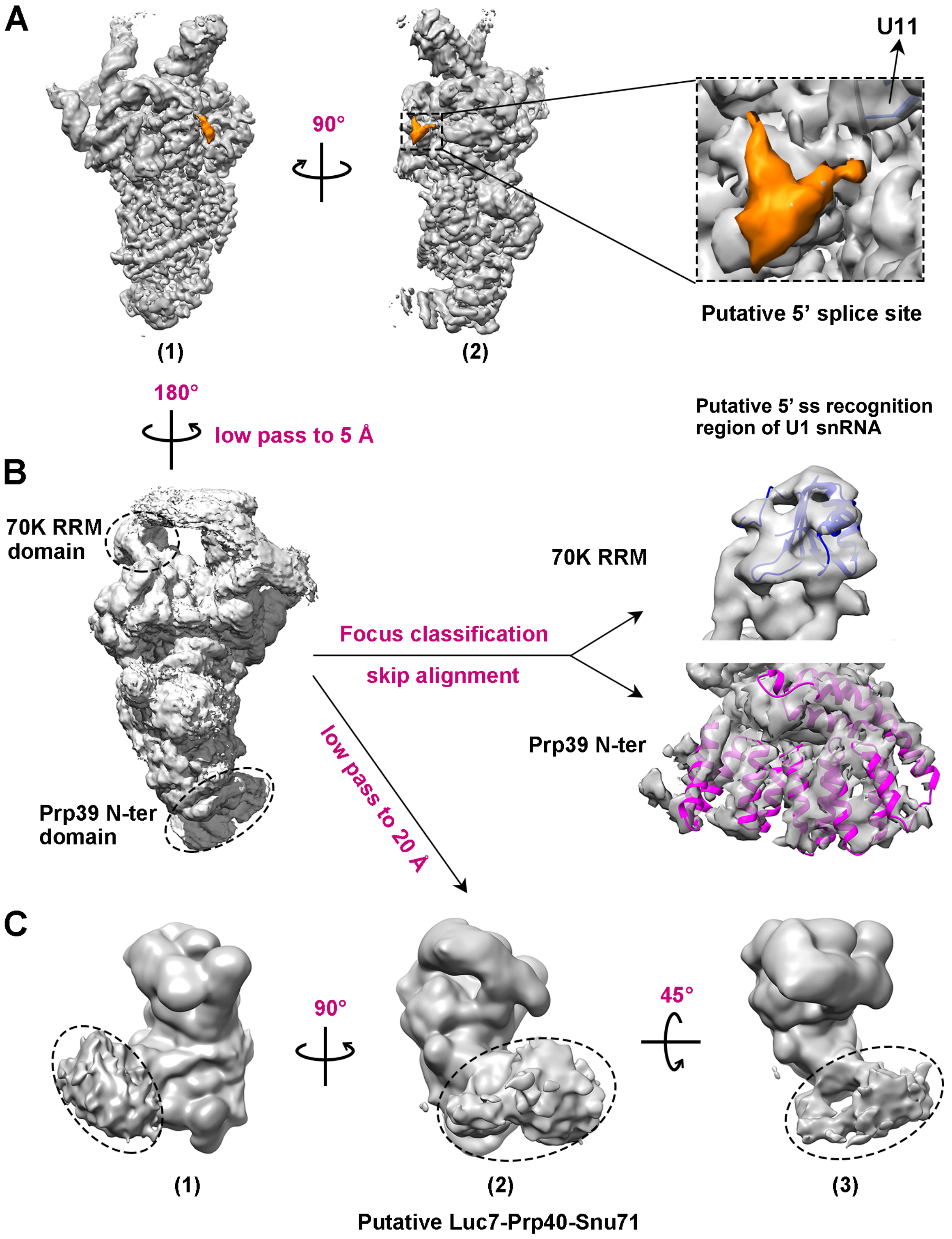

Supplementary Fig. 10. Representative flexible regions of the yeast U1 snRNP. (A) The final 3.6 Å map low-pass filtered at 5 Å showing additional density (yellow) near the 5' splice site. (B) Focus refinement results of U1-70K RRM domain and Prp39 N-terminal domain. The models were obtained from homology modeling using the I-TASSER server and were fitted into the map using Chimera. (C) The final 3.6 Å map low-pass filtered at 20 Å showing additional density near Luc7.

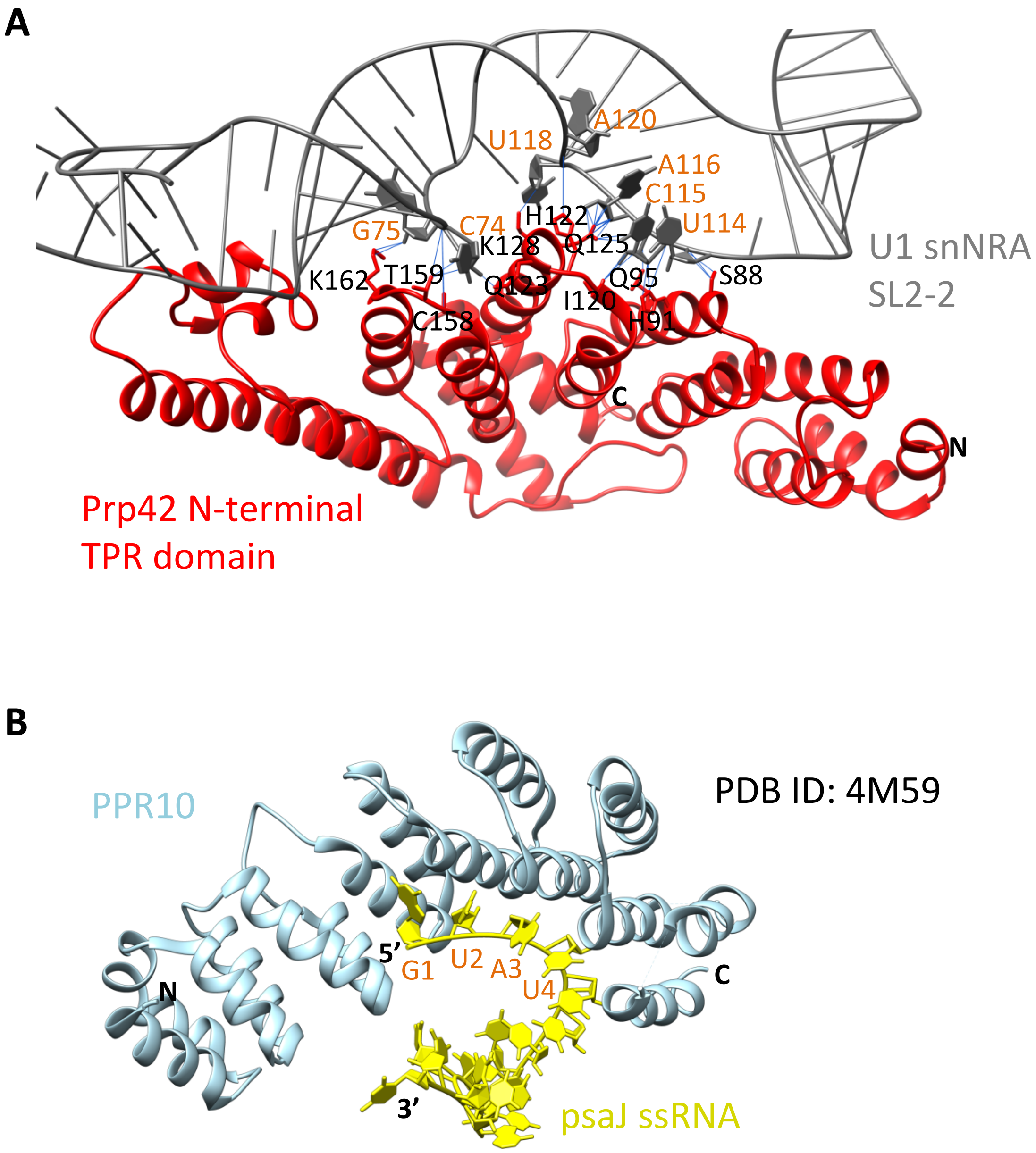

Supplementary Fig. 11. Illustration of the interaction mode between Prp42 and double stranded SL2-2 RNA (A) as well as the interaction between PPR10 and ssRNA (B).

Supplementary Fig. 12

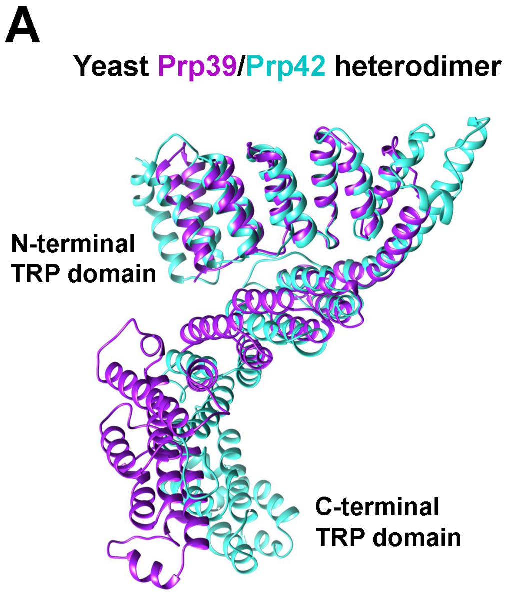

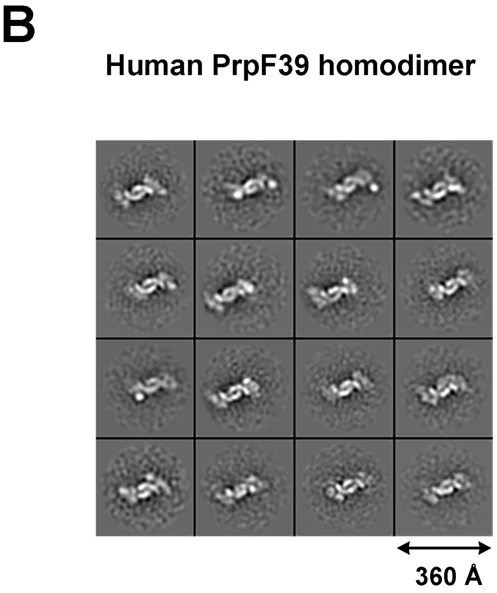

C

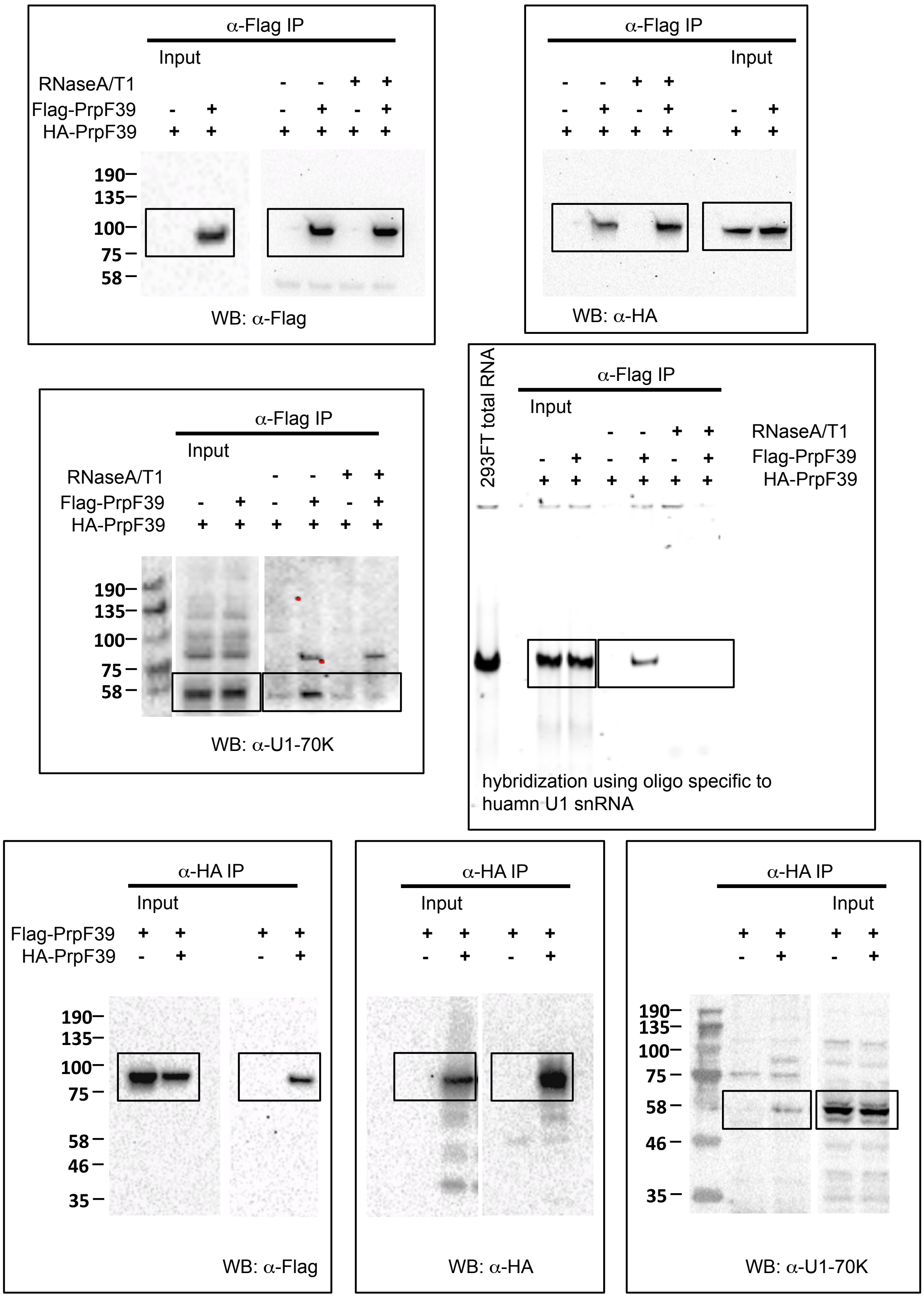

Supplementary Fig. 12. Structural and biochemical characterization of yeast Prp42, Prp39, and human PrpF39. (A) Prp42 and Prp39 have similar overall structures. When their N-terminal TPR domains are superimposed, their C-terminal TPR domains are only slightly shifted relative to each other. (B) Representative 2D classes of purified human PrpF39 examined by negative staining. (C) Uncropped scans of the Western blot and RNA gel in Fig. 4D.

Supplementary Table 1. Summary of protein and RNA components in yeast and human U1 snRNP.

Yeast U1 snRNP				Human U1 snRNP			Sequence similarity	
Protein name	Total number of residues	Residues /nucleotides present in the structural model	# of peptides identified in mass spectrometry[1]	Protein Name	Total number of residues	Residues /nucleotides present in the structural model [2]	Overall similarity in entire protein [3]	Similarity in the most homologous region (# of residues aligned) [4]
SmB/SmB1	196	3-64, 72-133	13	SmB	231	7-47, 65-87	35%	63% (96)
SmD1	146	1-119	1	SmD1	119	2-78	49%	49% (146)
SmD2	110	13-108	6	SmD2	118	11-76, 92-114	70%	75% (112)
SmD3	101	5-96	6	SmD3	126	6-81	58%	77% (86)
SmE/SmE1	94	10-62, 72-92		SmE	92	18-92	64%	71% (85)
SmF/SmX3	86	12-60, 65-83	1	SmF	86	6-81	53%	69% (71)
SmG/SmX2	77	2-76	2	SmG	76	4-76	68%	73% (73)
U1-70K /Snp1	300	1-91, 94-188 [5]	10	U1-70K	437	9-23, 63-89, 103-180	33%	52% (197)
U1C/Yhc1	231	3-134, 153-196	7	U1C	159	4-60	25%	71% (39)
U1A/Mud1	298	3-45, 55-125, 132-148	12	U1A	282	2-114 [6]	35%	54% (79)
Prp42	544	2-540	13	PRPF39 [7]	669		31%	42% (381)
Prp39	629	43-285 [5], 288-627	12	PRPF39	669		33%	42% (296)
Nam8	523	289-426, 432-448, 491-522	4	TIA-1	386		32%	44% (335)
Snu56	492	47-105, 111-169, 185-294	11	unknown				
Prp40	583	Not built	11	PRPF40 A<br>PRPF40 B	957<br>871		31%<br>30%	48% (467)<br>44% (559)
Luc7	261	39-84	8	Luc7L<br>Luc7L2<br>Luc7L3	371<br>392<br>432		34%<br>30%<br>29%	55% (234)<br>54% (234)<br>50% (246)
Snu71	620	Not built	25	RBM25	843		28%	59% (54)
U1 snRNA	568	11-25, 33-39, 41-97, 103-142, 149-175, 177-202, 236-289, 294-325, 516-565 [8]		U1 snRNA	164	2-164 [6]		

1. Although there is no peptide detected for SmE, it is likely a reflection of the low quantity of sample used for mass spectrometry analyses since SmE is clearly present in the cryoEM structure.
2. Numbers in this column refer to the 5.5Å structure of human U1 snRNP (PDB ID 3CW1) unless otherwise stated.
3. Sequence alignment and similarity calculation were performed using EMBOSS Needle (www.ebi.ac.uk/Tools/psa/emboss_needle/).
4. The most homologous region between the yeast and human proteins as well as sequence similarities in these regions are determined in BLAST.
5. The EM density for this region is of insufficient quality to build atomic models *de novo* and the homology model derived from I-TASSER is docked into the density as a rigid body using Chimera.
6. These numbers refer to the 4.4Å structure of human U1 snRNP (PDB ID 3PGW).
7. We propose that PrpF39 is the human homolog of yeast Prp42 based on sequence, structure, and biochemical analyses reported in this paper.
8. A total of 407nt representing 72% of the full-length yeast U1 snRNA is modeled.

Supplementary Table 2. Interacting residues on U1C and Prp42 identified by the "Find Contacts" function in Chimera using default parameters.

Residual on U1C	Interacting residual(s) on Prp42
HIS49	MET194
ARG59	SER219
LEU73	PRO224, TYR225
VAL75	ARG221, PRO224
THR76	GLN227
LYS90	ASP191
LEU97	ALA184, LEU185
ILE102	GLU145
THR104	HIS177
LEU105	LEU106, LEU107, GLU145
LEU108	PHE142, TYR263
TYR109	GLU71, LEU107
GLY111	TYR263
SER112	TYR263
PRO113	LEU70, THR265
GLY114	ASN72
TYR115	ASN72
LYS117	ASP76
VAL118	ILE75, THR265
PHE119	LEU107
ARG124	LEU79
PHE125	ASP76, LEU79, LEU80, LYS83, ILE44
ASP126	LEU79, TYR82
ASP157	ASN35, LYS39
THR159	GLU31
CYS160	LEU27, GLU31
PRO166	ILE261
ARG167	TYR260
PRO171	TYR260
LYS173	TYR260
LEU174	TYR252, PHE142
PRO176	TYR252
PRO177	HIS177, TYR252
LYS178	SER180
ILE179	SER180, TYR241, GLN245
LEU180	LEU187, TYR241
TRP183	THR237, LYS238, TYR241
THR186	MET242
ILE187	GLN245
PRO188	TYR246
PHE193	ILE254

Supplementary Table 3. Interacting nucleotides on U1 snRNA and residuals on Prp42 identified by the "Find Contacts" function in Chimera using default parameters.

Nucleotide on U1 snRNA	Interacting residual(s) on Prp42
SL2-2 C74	GLN123, THR159
SL2-2 G75	CYS158, THR159, LYS162
SL2-2 U114	SER88
SL2-2 C115	SER88, HIS91, ILE120, GLN125
SL2-2 A116	GLN125 GLN95
SL2-2 U118	LYS128
SL2-2 A120	HIS122
SL3-4 C253	ARG221
SL3-4 U254	MET194, ASP195, LEU196, ARG221, LYS222, GLY223
SL3-4 U256	MET194
SL3-4 U258	GLY223
SL3-4 C268	ARG221

Supplementary Table 4. BS3 crosslinked peptides identified using mass spectrometry in purified human PrpF39 proteins.

Peptide 1	Protein 1	Residues	Peptide 2	Protein 2	Residues	Local FDR [1]
[AVHGSLPIKMR] [2, 3]	PrpF39	496 to 506	[AVHGSLPIKMR]	PrpF39	496 to 506	0
[AVHGSLPIKMR]	PrpF39	496 to 506	[DKENTKLYLNLLE MEYSGDLK]	PrpF39	463 to 483	0
[EQDSLKR]	PrpF39	540 to 546	[AVHGSLPIKMR]	PrpF39	496 to 506	0
[HLFKIQK]	PrpF39	441 to 447	[SNNESSFYAVKLA R]	PrpF39	427 to 440	0
[KVLLEAIER]	PrpF39	454 to 462	[QNEENILNBFDKA VHGSLPIK]	PrpF39	484 to 504	0
[KVLLEAIER]	PrpF39	454 to 462	[HGNLEEAEHLLQD AIKNAK]	PrpF39	408 to 426	0
[NLPKSR]	PrpF39	448 to 453	[KVLLEAIER]	PrpF39	454 to 462	10%
[AVHGSLPIKMR]	PrpF39	496 to 506	[DKENTKLYLNLLE MEYSGDLK]	PrpF39	463 to 483	14%
[AVHGSLPIKMR]	PrpF39	496 to 506	[ENTKLYLNLLEME YSGDLK]	PrpF39	465 to 483	14%
[IQKNLPK] [3]	PrpF39	445 to 451	[IQKNLPK]	PrpF39	445 to 451	14%
[NLPKSR]	PrpF39	448 to 453	[HGNLEEAEHLLQD AIKNAK]	PrpF39	408 to 426	19%

1. FDR: False Discovery Rate.
2. Shaded amino acid indicates the residue that is crosslinked.
3. This peptide is crosslinked to itself through the same amino acid, which can only occur in self-associating oligomeric proteins.

Supplementary Table 5. BS3 crosslinked peptides identified using mass spectrometry in purified yeast U1 snRNP. Shaded residue is the reside that is crosslinked. All crosslinked residues identified for SmD1-U1-70K, SmD3-U1C, SmG-U1C, U1C-Prp42, and Prp42-Snu56 are between residues within 25 Å of each other, validating the crosslinking and mass spectrometry experiments.

Peptide 1	Protein 1	Residues	Peptide 2	Protein 2	Residues	Local FDR [1]
[LQELISKR] [2]	Luc7	169 to 176	[TKEEAEKEFITMLK]	Prp40	131 to 144	0%
[VPKNINLTGSFYLPK]	Snu56	151 to 165	[KVGNSEAFQSFDIWK]	Snu56	35 to 49	0%
[LQELISK]	Luc7	169 to 175	[RTKEEAEKEFITMLK]	Prp40	130 to 144	0%
[KNHSGAK]	Snu71	324 to 330	[HQMQKVFDQIR]	Snu71	313 to 323	0%
[ASKLPQR]	U1C	133 to 139	[STEPQLLK]	Prp42	47 to 54	0%
[VPKNINLTGSFYLPK]	Snu56	151 to 165	[KVGNSEAFQSFDIWK]	Snu56	35 to 49	0%
[NEKIQQK]	Prp40	479 to 485	[VDKEDISLIVDGLIKQR]	Prp40	462 to 478	0%
[GKNVINR]	SmD2	81 to 87	[ELWTEKK]	SmD2	74 to 80	0%
[MAFKEIGVHR]	U1-70K	163 to 172	[YFVKFGEIEK]	U1-70K	126 to 135	0%
[VPKNINLTGSFYLPK]	Snu56	151 to 165	[VGNSEAFQSFDIWKNLDR]	Snu56	36 to 53	0%
[VPKTQLDK]	SmB	53 to 60	[RPTDYPYAKR]	U1-70K	26 to 35	0%
[IKFDFK]	Snu71	498 to 503	[VGNSEAFQSFDIWKNLDR]	Snu56	36 to 53	0%
[KYMDK]	SmG	9 to 13	[FDIGDLVKASK]	U1C	125 to 135	0%
[IALTAGEKQEPGR]	Prp40	97 to 109	[HDTVSHAQVNGNR]	Prp40	84 to 96	0%
[LVNFLKK]	SmD1	3 to 9	[QIILPDSLNLDSLLVDQKQLNSLR]	SmD1	94 to 117	0%
[EVPIKIK]	Prp40	368 to 374	[FDKVDKEDISLIVDGLIK]	Prp40	459 to 476	0%
[LWMAPSEK]	Snu56	139 to 146	[VPKNINLTGSFYLPK]	Snu56	151 to 165	0%
[IKNAQLLDR]	U1-70K	79 to 87	[SGQIANDPSKK]	SmD1	119 to 129	0%
[DELLKK]	Prp42	206 to 211	[LKIDINYSGR]	Prp42	212 to 221	0%
[SGFIPILKNDLQR]	Snu71	26 to 38	[DIVFVSPQLYLSSQEGWKSDSAK]	Snu71	3 to 25	0%
[NLKTTR]	Prp39	210 to 215	[FFTSYKK]	Prp39	198 to 204	0%
[ASKLPQR]	U1C	133 to 139	[SAHHSFK]	U1C	144 to 150	0%
[YSHQKR]	Luc7	30 to 35	[BPQMHLTKHK]	Luc7	68 to 77	0%
[NEKIQQK]	Prp40	479 to 485	[VDKEDISLIVDGLIKQR]	Prp40	462 to 478	0%
[SLYLK]	Snu71	410 to 414	[WSDIYPHIKSDPR]	Prp40	379 to 391	0%
[DIINKHNHK]	U1C	44 to 52	[FIVVPDLLKNAPLFK]	SmD3	71 to 85	0%
[KYMDK]	SmG	9 to 13	[KSHLVGK]	U1C	22 to 28	0%
[ELFDKVWK]	Prp42	419 to 426	[KSGQEILLNNLVQFYSK]	Prp42	434 to 450	0%
[EKKMK]	Snu71	298 to 302	[HTAEERMKIQQVTEELDVLDVR]	Luc7	121 to 142	0%
[SGFIPILK]	Snu71	26 to 33	[STEPQLLKLIR]	Prp42	47 to 57	0%
[KTEIIK]	Prp42	344 to 349	[YQELKQFLPISLDQQIHTVSLQGVSSSFSR]	Snu71	99 to 128	9%
[LVNFLKK]	SmD1	3 to 9	[QIILPDSLNLDSLLVDQKQLNSLR]	SmD1	94 to 117	9%
[SDDIBAKK]	U1A	152 to 159	[RLENMKSQQENLK]	U1A	194 to 206	9%

[DLGLHDPKIBK]	Luc7	36 to 46	[QSLGKBPQMHLTK]	Luc7	63 to 75	9%
[WLTKK]	Prp39	299 to 303	[YLKFVTDPSK]	Prp39	259 to 268	11%
[FGEIEKIR]	U1-70K	130 to 137	[GYAFIVFKDPISSK]	U1-70K	149 to 162	11%
[KNAGQFIK]	Snu56	59 to 66	[GHIITSYR]	Snu56	172 to 179	11%
[ELAPIQLSDGK]	Snu71	451 to 461	[AISAGKAAAITLPEGTVK]	Snu71	462 to 479	11%
[YEDIKLSK]	U1-70K	71 to 78	[SGQIANDPSKK]	SmD1	119 to 129	13%
[TFBQNSLYSSR]	Prp42	396 to 406	[KVGNSEAFQSFDIWK]	Snu56	35 to 49	13%
[KVRMGKAR]	U1A	105 to 112	[VLTHEDVLNEYLKIVNTIEN DLQNK]	Prp40	319 to 343	15%
[KEKKQAQK]	SmB	114 to 121	[YQELKQFLPISLDQQIHTVS LQGVSSSFSR]	Snu71	99 to 128	14%
[IALTAGEKQEPGR]	Prp40	97 to 109	[TINEEESQYANNSKLLNVR]	Prp40	110 to 128	14%
[KYMDK]	SmG	9 to 13	[ASKLPQR]	U1C	133 to 139	14%
{mFNRK]	NCBP1	0 to 5	[NNQGYBFVDFPSSTHAANA LLK]	Nam8	104 to 125	13%
[YEDIKLSK]	U1-70K	71 to 78	[IKNAQLLDR]	U1-70K	79 to 87	17%
[FKEAFQK]	Prp40	204 to 210	[HSVVNEKTK]	Prp40	239 to 247	17%
[GKNVINR]	SmD2	81 to 87	[ELWTEKK]	SmD2	74 to 80	17%
[LRKEK]	SmB	125 to 129	[LNSNGIAmASLYLTGGQQP TASDNIASLQYINIR]	SmD1	55 to 88	18%
[ELWTEK]	SmD2	74 to 79	[KGKNVINR]	SmD2	80 to 87	18%
[DELLKK]	Prp42	206 to 211	[LKIDINYSGR]	Prp42	212 to 221	20%
[IKNQLGSTYK]	U1A	277 to 286	[AKVSNTMENPPNK]	U1A	215 to 227	20%

1. FDR: False Discovery Rate.
2. Shaded amino acid indicates the residue that is crosslinked.

Supplementary Table 6. CryoEM data collection and refinement statistics.

**Data collection**	
EM equipment	FEI Titan Krios
Voltage (KV)	300
Detector	Gatan K2
Pixel size (Å)	1.36
Electron dose (e^-/Å^2)	51.9
Defocus range (µm)	-1.6 ~ -4.0
**Reconstruction**	
Software	RELION2-beta
Number of used particles	352,900
Accuracy of rotation (°)	2.10
Accuracy of translation (pixels)	0.86
Map sharpening B-factors (Å^2)	-137.7
Final Resolution (Å)	3.6
**Model building**	
Software	COOT
**Refinement**	
Software	PHENIX & REFMAC
Resolution	3.6
Average Fourier shell correlation	0.825
R-factor	0.272
**Model composition**	
Protein residues	2696
RNA nucleotides	308
**Validation**	
R.m.s deviations	
Bonds length (Å)	0.008
Bonds angle (°)	1.220
Ramachandran plot statistics (%)	
Preferred	92.09
Allowed	7.27
Outlier	0.64
Molprobity score	2.05
Clashscore	3.24
Rotamers outliers (%)	3.90

Supplementary Table 7. Model validation summary for individual proteins of yeast U1 snRNP (all *de novo* atomic models are shown here).

**Molecule**	**Molprobity Scores**	Ramachandran plot statistics (%)			**EMRinger* Score**
		Preferred	Allowed	Outlier	
Prp42	2.21	91.99	7.27	0.74	3.90
Prp39	1.58	93.49	6.51	0.00	3.21
Nam8	1.61	92.82	6.63	0.55	2.70
Snu56	1.62	93.30	6.70	0.00	3.72
Luc7	2.31	88.64	9.09	2.27	2.18
U1-70K	1.44	93.26	4.49	2.25	3.11
U1C	1.78	88.95	10.47	0.58	3.68
U1A	1.55	89.60	7.20	3.20	1.75
Sm ring	2.05	92.20	7.48	0.32	3.17

*EMRinger: a side-chain-directed approach to study model-to-map agreement in cryoEM. Refinement should result in EMRinger scores above 1.0 for well-refined structures with maps in 3- to 4- Å range.

Supplementary Table 8. Summary of model building, refinement and validation for yeast U1 snRNA.

**Model building**	
Software	COOT & RCrane
**Refinement**	
Software	REFMAC
**Validation**	
Clash scores	4.78
Correct sugar puckers (%)	97.73
Good backbone (%)	77.92
Good bonds (%)	100.00
Good angles (%)	99.81
