# Supplementary material for: CryoEM structure of Saccharomyces cerevisiae U1 snRNP offers insight into alternative splicing
Source: Nat Commun. 2017 Oct 19;8:1035. doi: 10.1038/s41467-017-01241-9 (PMC5648754; doi:10.1038/s41467-017-01241-9)
Supplement: Supplementary file 2 — Description of Additional Supplementary Files [file 41467_2017_1241_MOESM2_ESM.docx]

**Description of Additional Supplementary Files**

File Name: Supplementary Movie 1: cryoEM structure of yeast U1 snRNP

Description: Cryo EM structure of S. cerevisiae U1 snRNP. This movie describes the overall structure as well as the density and atomic model of each individual component.
